# Prenyleudesmanes and A Hexanorlanostane from the Roots of *Lonicera macranthoides*

**DOI:** 10.3390/molecules24234276

**Published:** 2019-11-23

**Authors:** Hui Lyu, Wenjuan Liu, Bai Bai, Yu Shan, Christian Paetz, Xu Feng, Yu Chen

**Affiliations:** 1 Jiangsu Key Laboratory for the Research and Utilization of Plant Resources, The Jiangsu Provincial Platform for Conservation and Utilization of Agricultural Germplasm, Institute of Botany, Jiangsu Province and Chinese Academy of Sciences, Nanjing 210000, China; hlyu@ice.mpg.de (H.L.); baibai0924@126.com (B.B.); shanyu79@126.com (Y.S.); 2 Max Planck Institute for Chemical Ecology, D-07745 Jena, Germany; cpaetz@ice.mpg.de; 3 Naval Compound Community Health Care Station, Beijing 100853, China; wenjuan9718@sina.com

**Keywords:** *Lonicera macranthoides*, Caprifoliaceae, Prenyleudesmanes, Hexanortriterpenes, Antiproliferative

## Abstract

Three previously undescribed compounds, two prenyleudesmanes (**1** and **2**), and one hexanorlanostane (**3**), were isolated from the roots of *Lonicera macranthoides*. Their structures were established based on 1D and 2D nuclear magnetic resonance (NMR) spectra and high-resolution electrospray ionization mass spectral (HR-ESI-MS) data. The absolute configurations of **1** and **3** were determined by X-ray diffraction. To the best of our knowledge, this is the first time that the absolute configuration of a prenyleudesmane with a *trans*-decalin system and a hexanorlanostane have been unambiguously confirmed by single-crystal X-ray diffraction with Cu Kα radiation. Thecompounds were tested for their antiproliferative activity on the cancer cell lines (HepG2 and HeLa). The compounds **1**–**3** exhibited moderate inhibitory effects against two human cancer cell lines.

## 1. Introduction

*Lonicera macranthoides* Hand.-Mazz., a plant of the genus *Lonicera* in the family Caprifoliaceae, is mainly distributed in the southwest of China [1]. The dried flower buds of *L. macranthoides* are commonly used as a raw material in traditional Chinese medicine for treating fever, inflammation, and infectious diseases [2]. Earlier phytochemical studies on the plant have shown the presence of various triterpenoid saponins (e.g., hederagenin saponins, oleanolic acid saponins, 18-oleanene saponins, and lupane saponins) [3,4,5,6], flavonoids [7], phenolic acids [8,9], and iridoids [8,9] in aerial parts and flowers of the plant. Because of our studies of *L. macranthoides*, we became interested in the diterpenes of this species. Recently, we reported the first known occurrence of diterpenes (e.g., labdane, aphidicolane, and *syn*-pimarane) in the roots of *L. macranthoides* [10,11,12]. To explore further unknown diterpenes, we reinvestigated the roots of *L. macranthoides*. Here, we report on the isolation and characterization of two new diterpenes, lonimacranthoidin C (**1**) and lonimacranthoidin D (**2**), and a novel hexanorlanostane, lonimacranthoidin E (**3**). Compounds **1**–**3** were screened for antiproliferative activity against two human cancer cell lines, HepG2 and HeLa.

## 2. Results and Discussion

**Figure 1:**
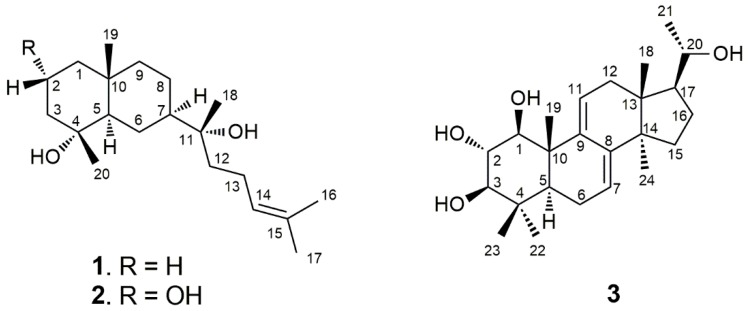
Chemical structures of the compounds **1**–**3**.

An ethanolic extract of dried roots of *L. macranthoides* was suspended in water and partitioned sequentially between petroleum ether and ethyl acetate (EtOAc). The EtOAc fraction was subjected to repeated separation by column chromatography (CC) over silica gel and Sephadex LH-20. Selected fractions were further purified by preparative HPLC to yield three pure compounds, including the two prenyleudesmanes (**1**, **2**) and a hexanorlanostane (**3**), as seen in Figure 1. The structure elucidation was carried out by high-resolution mass spectrometry (HRMS), nuclear magnetic resonance (NMR) spectroscopy (^1^H NMR, ^13^C NMR, ^1^H-^1^H homonuclear chemical shift correlation spectroscopy (COSY), ^1^H-^13^C heteronuclear single quantum coherence (HSQC), ^1^H-^13^C heteronuclear multiple bond correlation (HMBC), and ^1^H-^1^H rotating frame Overhauser effect spectroscopy (ROESY)), and single-crystal X-ray diffraction analysis.

**Figure 2:**
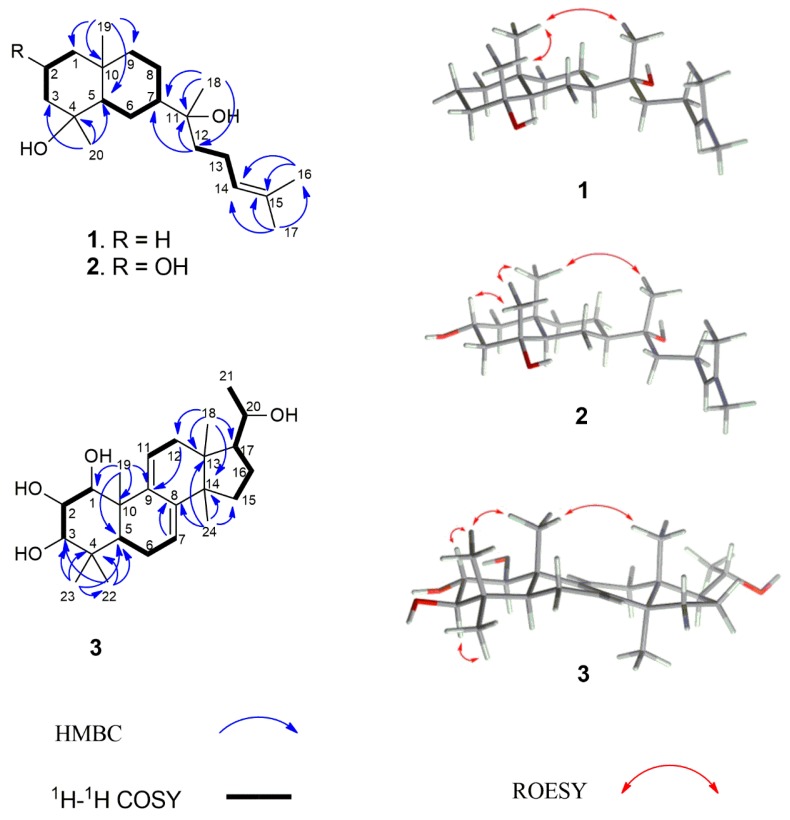
Key 2D NMR correlations of compounds **1**, **2**, and **3**.

**Figure 3:**
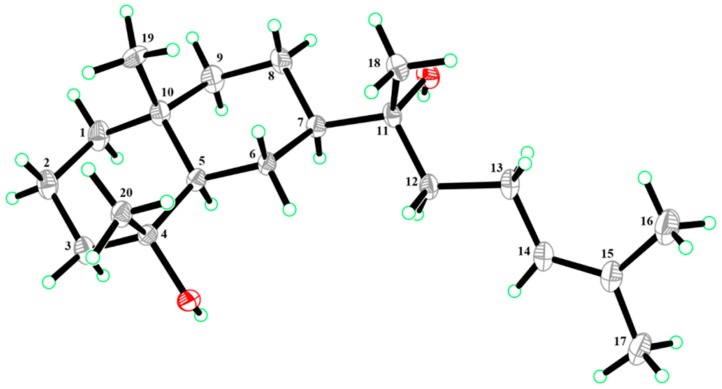
X-ray Oak Ridge thermal-ellipsoid plot program (ORTEP) drawing of compound **1**.

Compound **1** was obtained as colorless crystals. The molecular formula of C_20_H_36_O_2_Na was determined by the pseudomolecular ion peak at *m*/*z* 331.2607 [M + Na]^+^ (calculated: 331.2608) in positive HR-ESI-MS, corresponding to three unsaturations. The UV spectrum showed the absorption maximum at λ_max_ 201 nm. The compound showed a positive optical rotation of [α${]}_{D}^{25}$+26.4 (c 0.100 in methanol). The ^1^H NMR spectrum of **1** displayed signals for one olefinic proton at *δ*_H_ 5.14 (1H, dd, *J* = 7.0/7.0, H-14), five methyl singlets at *δ*_H_ 0.86 (3H, s, H_3_-19), *δ*_H_ 1.11 (3H, s, H_3_-20), *δ*_H_ 1.16 (3H, s, H_3_-18), *δ*_H_ 1.63 (3H, s, H_3_-17), and *δ*_H_ 1.69 (3H, s, H_3_-16), and overlapping aliphatic methylene and/or methine signals (*δ*_H_ 1.05–2.04). The assignment of the latter could be accomplished by a series of selective total correlation spectroscopy (SELTOCSY) experiments (see Supporting Information). The ^13^C NMR and distortionless enhancement by polarization transfer (DEPT) NMR spectra of **1** showed the presence of 20 carbon resonances, including two signals of olefinic carbons at *δ*_C_ 124.6 (C-14) and *δ*_C_ 131.6 (C-15), five signals for methyl groups at *δ*_C_ 17.6 (C-16), *δ*_C_ 18.7 (C-19), *δ*_C_ 22.6 (C-20), *δ*_C_ 24.1 (C-18), and *δ*_C_ 25.7 (C-17), eight methylene signals at *δ*_C_ 41.0 (C-1), *δ*_C_ 20.1 (C-2), *δ*_C_ 43.5 (C-3), *δ*_C_ 21.4 (C-6), *δ*_C_ 21.8 (C-8), *δ*_C_ 44.6 (C-9), *δ*_C_ 39.7 (C-12), and *δ*_C_ 22.3 (C-13), two methine signals at *δ*_C_ 55.0 (C-5) and *δ*_C_ 48.3 (C-7), one signal for a quaternary carbon at *δ*_C_ 34.6 (C-10), and two signals of oxygenated tertiary carbons at *δ*_C_ 72.3 (C-4) and *δ*_C_ 74.5 (C-11). The interpretation of NMR spectra and the degree of unsaturation deduced from HRMS data suggested that compound **1** was a bicyclic diterpene possessing a trisubstituted double bond and two hydroxyl groups (OH-4 and OH-11). Analysis of the ^1^H-^1^H COSY and HSQC spectra of **1** provided three partial structures shown by bold lines in Figure 2. The interpretation of the HMBC spectrum of **1** showed correlations from H_3_-17 to C-14, C-15, and C-16, and from H_3_-16 to C-14, C-15, and C-17; these enabled the localization of the double bond at C-14. This spin system is further characterized by a coupling of H-12 to H-14. HMBC correlations from H-12 to C-11, and from H_3_-18 to C-11 and C-12, eventually resulted in the definition of a side chain of eight carbons, including a trisubstituted double bond (Δ^14^). A further HMBC correlation from H-12 to C-7 and from H_3_-18 to C-7 indicated the linkage to C-7 of the decalin ring system. The two hydroxylated positions at C-4 and C-11, respectively, could be confirmed by the HMBC correlations from H_3_-20 to C-3, C-4, and C-5, and from H_3_-18 to C-11 and C-12, as seen in Figure 2. In summary, the NMR data analysis, as seen in Table 1, revealed a prenyleudesmane skeleton similar to dysokusone A [13], and the structure of **1** was determined as shown in Figure 1. The relative configuration of **1** was partially established by analyzing its ROESY correlations. Nuclear Overhauser effect (NOE) correlations between H_3_-18, H_3_-19, and H_3_-20 suggested a cofacial arrangement. Finally, crystals of compound **1** were obtained and subjected to X-ray diffraction analysis, as seen in Figure 3. The absolute configuration of **1** was determined as (4*R*,5*R*,7*R*,10*R*)-4,10-dimethyl-7-(11*R*-hydroxy-11,15-dimethyl-14-ene-11-yl)-*trans*-decalin-4-ol by Cu X-ray crystallography (Flack parameter = −0.05 (11), Figure 3) [14,15] and named as lonimacranthoidin C (**1**). Ours was the first successful single-crystal X-ray analysis of a prenyleudesmane with a *trans*-decalin scaffold.

Compound **2** was obtained as milky oil with the molecular formula C_20_H_36_O_3_ as determined by HR-ESI-MS (*m*/*z* 347.2551 [M + Na]^+^, calculated: *m*/*z* 347.2557 for [C_20_H_36_O_3_ + Na]^+^). Similar to **1**, compound **2** showed three unsaturations. The UV spectrum of **2** showed an absorption at λ_max_ 201 nm and a positive optical rotation of [α${]}_{D}^{25}$ + 10.0 (c 0.100 in methanol) was determined. The assignment of all proton and carbon chemical shifts of **2** was achieved by analyzing the 1D and 2D NMR spectra, as seen in Table 1. Similar to **1**, the structure of **2** was also elucidated as a prenyleudesmane-type diterpene. Unlike the chemical shifts of position 2 in **1** (*δ*_C_ 20.1 and *δ*_H_ 1.56/1.58 (H-2*β*/H-2*α*, respectively)), the corresponding structural elements in **2** were an oxygenated methylene (*δ*_C_ 68.2) and a hydroxymethine *(δ*_H_ 4.28, H-2). Thus, **2** was determined as the C-2 hydroxylated derivative of **1,** as shown in Figure 1. The stereochemistry at C-2 was established by the occurrence of NOE correlations between H_3_-19, H_3_-20 H_3_-18, and H-2 which indicated a cofacial orientation. Hence, the absolute configuration for C-2 was assigned, based on the X-ray determined configuration of **1,** as *R*-configured. Due to the occurrence of similar chemical shifts for C-4, C-5, C-7, C-10, and C-11, as seen in Table 1, similar NOESY correlations, as seen in Figure 2, similar values for the optical rotation, and the above defined configuration of C2, compound **2** was determined as (2*R*,4*R*,5*R*,7*R*,10*R*)-4,10-dimethyl-7-(11*R*-hydroxy-11,15-dimethyl-14-ene-11-yl)-*trans*-decalin-2,4-diol, and named lonimacranthoidin D (**2**). 

**Figure 4:**
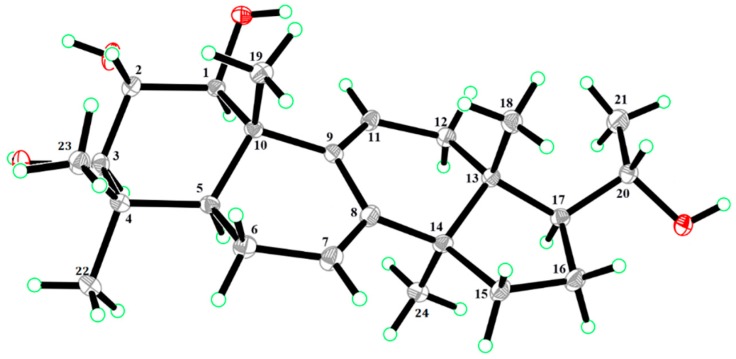
X-ray ORTEP drawing of compound **3**.

The molecular formula of compound **3** was assigned as C_24_H_38_O_4_ by its positive HR-ESI-MS data (*m*/*z* 413.2667, [M + Na]^+^; calculated: 413.2662), which indicated six unsaturations in the molecule. Compound **3** was obtained as colorless crystals with an UV spectrum having an absorption maximum at λ_max_ 243 nm. The compound showed a positive optical rotation of [α${]}_{D}^{25}$ + 12.6 (c 0.100 in methanol). The ^1^H NMR spectrum of **3** showed resonances of two olefinic protons at *δ*_H_ 6.31 (1H, d, *J* = 6.0 Hz, H-11) and *δ*_H_ 5.49 (1H, d, *J* = 5.0 Hz, H-7), six methyl resonances at *δ*_H_ 0.57 (3H, s, H_3_-18), *δ*_H_ 1.06 (3H, s, H_3_-19), *δ*_H_ 1.21 (3H, d, *J* = 6.2 Hz, H_3_-21), *δ*_H_ 0.90 (3H, s, H_3_-22), *δ*_H_ 1.02 (3H, s, H_3_-23), and *δ*_H_ 0.91 (3H, s, H_3_-24), four hydroxymethines at *δ*_H_ 3.57 (1H, d, *J* = 10.0 Hz, H-1), *δ*_H_ 3.48 (1H, dd, *J* = 10.0/10.0 Hz, H-2), *δ*_H_ 3.04 (1H, d, *J* = 10.0 Hz, H-3), and *δ*_H_ 3.62 (1H, dd, *J* = 6.2/9.0 Hz, H-20), and further overlapping aliphatic methylenes and/or methines in the range *δ*_H_ 1.10 to *δ*_H_ 2.30, which were assigned by means of SELTOCSY experiments (see Supporting Information). The ^13^C NMR and DEPT spectra of **3** showed the presence of four olefinic carbons at *δ*_C_ 119.1 (C-7), *δ*_C_ 144.0 (C-8), *δ*_C_ 142.1 (C-9), and *δ*_C_ 119.1 (C-11). The remaining four MS-predicted unsaturations were assigned to a tetracyclic ring system. In addition, six methyl groups at *δ*_C_ 15.4 (C-18), *δ*_C_ 15.9 (C-19), *δ*_C_ 22.4 (C-21), *δ*_C_ 15.9 (C-22), *δ*_C_ 27.6 (C-23), and *δ*_C_ 24.9 (C-24), four methylenes at *δ*_C_ 22.4 (C-6), *δ*_C_ 36.4 (C-12), *δ*_C_ 31.1 (C-15), and *δ*_C_ 25.8 (C-16), two methines at *δ*_C_ 47.1 (C-5) and *δ*_C_ 53.0 (C-17), four oxygenated methines at *δ*_C_ 78.4 (C-1), *δ*_C_ 72.9 (C-2), *δ*_C_ 78.7 (C-3), and *δ*_C_ 70.4 (C-20), and four quaternary carbons at *δ*_C_ 38.1 (C-4), *δ*_C_ 43.2 (C-10), *δ*_C_ 42.0 (C-13), and *δ*_C_ 49.9 (C-14) were observed. Thus, **3** was assigned as a hexanortriterpene derivative with two trisubstituted double bonds (Δ^7^ and Δ^9^) and four hydroxyl groups (OH-1, OH-2, OH-3, and OH-20). Interpretation of the ^1^H-^1^H COSY data resulted in the identification of four spin systems: H-1/H-2/H-3, H-5/H-6/H-7, H-11/H-12, and H-15/H-16/H-17/H-20/H-21. The HMBC correlations from H_3_-18 to C-12, C-13, C-14, and C-17; from H_3_-19 to C-1, C-5, C-9, and C-10; from H_3_-22 to C-3, C-4, C-5, and C-23; from H_3_-23 to C-3, C-4, C-5, and C-22; and from H_3_-24 to C-8, C-13, C-14, and C-15 allowed for the positioning of two methyl groups at C-4 and four methyl groups at C-10, C-13, C-14, and C-20, respectively. These data indicated that compound **3** was an unusual hexanorlanostane that had been described earlier as aglycon, from the saponins of the sea cucumber *Cucumaria koraiensis* [16,17]. The full assignment of all positions in the molecule was accomplished by the interpretation of the HSQC and HMBC data, as seen in Table 1, suggesting that **3** was 1,2,3,20-tetrahydroxy-hexanorlanostan-7, 9(11)-diene, as shown in Figure 1. In the ROESY spectrum of **3**, correlations between H_3_-18/H_3_-19/H_3_-23/H-2 and H_3_-22/H-3 indicated that H_3_-18, H_3_-19, H_3_-23, and H-2 were on one side of the molecular plane, while H_3_-22 and H-3 were located on the opposite side. Fortunately, crystals of compound **3** could be obtained and were subjected to single-crystal X-ray diffraction analysis, as seen in Figure 4. Based on our results, the absolute configuration of **3** was determined as (1*S*,2*S*,3*R*,5*S*,10*S*,13*R*,14*R*,17*S*,20*S*)-1,2,3,20-tetrahydroxy-hexanorlanostan-7, 9(11)-diene (**3**) by Cu X-ray crystallography (Flack parameter = 0.20 (6), Figure 4) and named lonimacranthoidin E.

Compounds **1**–**3** were furthermore tested for their antiproliferative effect on the Human Hepatocellular Carcinoma Cell lines (HepG2), Human Cervical Carcinoma Cell line (HeLa), and the Human Aortic Smooth Muscle Cell line (HASMC). The results, as seen in Table 2, demonstrated that **1**–**3** showed moderate antiproliferative activities (IC_50_ 12.5 ± 0.9 to 64.9 ± 3.5 μM) against the two tumor cell lines. No significant effect against HASMC was observed.

Prenyleudesmanes are a rare class of diterpenes that were originally isolated from marine algae [18,19] and marine mollusks [20,21,22,23,24]. Prenyleudesmanes were also found in fungi [25] and plants of the genus *Dysoxylum* [13,26,27,28]. Our report on the isolation and full structure elucidation of lonimacranthoidin C (**1**) and lonimacranthoidin D (**2**) from *L. macranthoides* therefore suggests another source for prenyleudesmanes in nature. Interestingly, the hexanorlanostane **3** was initially found in sea creatures [16,17]. Lonimacranthoidin E (**3**) is the first example of a hexanorlanostane isolated from a terrestrial plant.

## 3. Materials and Methods

### 3.1. General Experimental Procedures

Thin-layer chromatography was carried out on silica gel 60 GF254 (Merck) plates. Preparative HPLC (LC-20AR, Shimadzu, Kyoto, Japan) was conducted on a Shim-pack GIS C_18_ column (5 μm, 250 × 20 mm, Shimadzu). Column chromatography (CC) was performed on silica gel (200–300 mesh) and Sephadex LH-20. LC-HRMS spectra were obtained from an Agilent 1260 UPLC-DAD-6530 ESI Q-TOF MS (Agilent Technologies GmbH, Waldbronn, Germany). Optical rotation values were measured using a Jasco P-1020 polarimeter. NMR data were obtained using a Bruker Avance 500 MHz or Bruker Avance 300 MHz spectrometers (Bruker Biospin GmbH, Karlsruhe, Germany). Tetramethylsilane was used as an internal standard. The X-ray structures were solved by direct methods (SHELXL-97). The X-ray crystallographic data were collected on a Bruker SMART APEX-II CCD diffractometer using graphite monochromatic Cu K_α_ radiation. 

### 3.2. Plant Material

The roots of *L. macranthoides* were collected from Longhui in the Hunan province of China in July 2015. The plants were taxonomically identified by Professor Changqi Yuan (Institute of Botany, Jiangsu province and Chinese Academy of Sciences). A voucher specimen (No. 20150701) has been deposited in the herbarium of the Institute of Botany, Jiangsu province, and Chinese Academy of Sciences.

### 3.3. Extraction and Isolation

The dried roots (4.0 kg) of *L. macranthoides* were milled and repeatedly extracted with 95% EtOH for 2 h under reflux (80 °C). After evaporation in vacuo, the crude extract (472.6 g) was resuspended in H_2_O and partitioned with petroleum ether and ethyl acetate (EtOAc), in succession. The EtOAc fraction (94 g) was subjected to column chromatography (silica gel, CH_2_Cl_2_-MeOH 100:0–0:100) to produce six fractions (F1-F6) on the basis of TLC analysis. F2 (21 g) was purified by column chromatography on Sephadex LH-20 (CH_2_Cl_2_-MeOH, 1:1), followed by preparative HPLC using MeOH-H_2_O (65:35, *v*/*v*, flow rate 1.0 mL/min) as an eluent to obtain the compounds **1** (11.0 mg) and **2** (6.0 mg). F5 (11 g) was purified by preparative HPLC with MeOH–H_2_O (55:45, *v*/*v*, flow rate 3.0 mL/min) as an eluent to obtain compound **3** (13.0 mg).

### 3.4. Compound Characterization 

Lonimacranthoidin C (**1**): colorless crystals (MeOH); [α${]}_{D}^{25}$ + 26.4 (c 0.100 in MeOH); UV (MeOH) λ_max_ 201 nm; mp 101–103 °C; HR-ESI-MS *m*/*z* 331.2607 [M + Na]^+^ (calculated for C_20_H_36_O_2_: 331.2608); ^1^H NMR (500 MHz, CDCl_3_) and ^13^C NMR (125 MHz, CDCl_3_) spectroscopic data, see Table 1. Lonimacranthoidin C (**1**) was recrystallized in methanol/ethyl acetate (3:1). A single-crystal X-ray diffraction analysis using Cu Kα radiation (1.54178 Å) was carried out to confirm the structure. *M* = 308.49, monoclinic, P2_1_ 2_1_ 2_1_, *a* = 11.5498 (17) Å, *b* = 12.2435 (14) Å, *c* = 27.246 (3) Å, *α* = *γ* = *β* = 90.00°, *V* = 3852.8 (9) Å^3^, *Z* = 8, *Dc* = 1.064 mg mm^−3^, *T* = 153 (2) K, *F* (000) = 1376.0. The crystallographic data centre has assigned the code Cambridge Crystallographic Data Centre (CCDC) 1,941,729 for the crystal structure of lonimacranthoidin C (**1**). The CCDC contains the supplementary crystallographic data for this paper. These data can be obtained free of charge via http://www.ccdc.cam.ac.uk/conts/retrieving.html.

Lonimacranthoidin D (**2**): milky oil (MeOH); [α${]}_{D}^{25}$ + 10.0 (c 0.100 in MeOH); UV (MeOH) λ_max_ 201 nm; HR-ESI-MS *m*/*z* 347.2551 [M + Na]^+^ (calculated for C_20_H_36_O_3_, 347.2557); ^1^H NMR (500 MHz, CDCl_3_) and ^13^C NMR (125 MHz, CDCl_3_) spectroscopic data are listed in Table 1.

Lonimacranthoidin E (**3**): colorless crystals (MeOH); [α${]}_{D}^{25}$ + 12.6 (c 0.100 in MeOH); UV (MeOH) λ_max_ 243 nm; mp 244–246 °C; HR-ESI-MS *m*/*z* 413.2667 [M + Na]^+^ (calculated for C_24_H_38_O_4_: 413.2662); ^1^H NMR (300 MHz, in CDCl_3_:CD_3_OD = 1:1) and ^13^C NMR (75 MHz, in CDCl_3_:CD_3_OD = 1:1), for NMR spectroscopic data, see Table 1. Lonimacranthoidin E (**3**) was re-crystallized in methanol/ethyl acetate (1:1). A single-crystal X-ray diffraction analysis using Cu Kα radiation (1.54178 Å) was carried out to confirm the structure. *M* = 390.54, monoclinic, P2_1_ 2_1_ 2_1_, *a* = 6.0714 (4) Å, *b* = 13.2797 (8) Å, *c* = 28.6232 (17) Å, *α* = *γ* = *β* = 90.00°, *V* = 2307.87 (2) Å^3^, *Z* = 4, *Dc* = 1.124 mg mm^−3^, *T* = 153 (2) K, *F* (000) = 856. The crystallographic data centre has assigned the code CCDC 1,941,730 for the crystal structure of lonimacranthoidin E (**3**) The CCDC contains the supplementary crystallographic data for this paper. These data can be obtained free of charge via http://www.ccdc.cam.ac.uk/conts/retrieving.html.

### 3.5. Biological Assay 

HepG2, HeLa, and HASMC cell lines were cultured in Dulbecco’s modified Eagle’s medium (Gibco, Grand Island, NY, USA) supplemented with 10% fetal bovine serum (Gibco), 100 μg/mL penicillin, and 100 μg/mL streptomycin. The cells were cultivated in a humidified atmosphere of 5% CO_2_ at 37 °C. Antiproliferative assays of the compounds **1**–**3** against the above-mentioned three cell lines were evaluated using the 3-(4,5-dimethylthiazol-2-yl)-2,5-diphenyltetrazoliumbromide (MTT) assay, carried out according to protocols [29] described previously.

## Figures and Tables

**Table 1:** ^1^H and ^13^C NMR spectral data of compounds **1**, **2,** and **3**.

Atom	1 ^a, c^		2 ^a,c^		3 ^b,c^	
	δ_C_	δ_H_	δ_C_	δ_H_	δ_C_	δ_H_
**1α**	41.0	1.07 ddd 12.5/12.5/6.0	46.7	1.34 dd 14.0/3.2	78.4	3.57 d 10.0
**1β**		1.38 d 12.5		1.67 m		
**2α**	20.1	1.58 m	68.2	4.28 ddd 7.0/3.0/3.0	72.9	3.48 dd 10.0/10.0
**2β**		1.56 m				
**3α**	43.5	1.35 ddd 12.5/12.5/5.5	48.5	1.66 m	78.7	3.04 d 10.0
**3β**		1.79 d 12.5		2.01 d 14.0		
**4**	72.3		71.6		38.1	
**5**	55.0	1.19 d 12.5	54.4	1.29 dd 12.5/2.0	47.1	1.16 dd 5.0/12.0
**6α**	21.4	1.86 d 12.5	21.2	1.87 d 12.5	22.4	2.20 dd 12.0/17.0
**6β**		1.03 ddd 12.5/12.5/12.5		1.14 ddd 12.5/12.5/12.5		2.15 dd 5.0/5.0/17.0
**7**	48.3	1.41 dddd 12.5/12.5/3.0/3.0	48.5	1.42 dddd 12.5/12.5/3.0/3.0	119.5	5.49 d 5.0
**8α**	21.8	1.59 d 12.5	21.2	1.56 d 12.5	142.1	
**8β**		1.33 dddd 12.5/12.5/12.5/3.0		1.37 dddd 12.5/12.5/12.5/3.5		
**9α**	44.6	1.45 ddd 12.5/3.0/3.0	44.9	1.49 ddd 12.5/3.0/3.0	144.0	
**9β**		1.15 ddd 12.5/12.5/3.0		1.12 m		
**10**	34.6		34.0		43.2	
**11**	74.5		74.6		119.1	6.31 d 6.0
**12α**	39.7	1.53 dd 8.2/8.2	39.5	1.52 dd 8.1/8.1	36.4	2.18 d 17.0
**12β**						2.06 d 6.0/17.0
**13**	22.3	2.06 m	22.3	2.04 m	42.0	
**14**	124.6	5.14 dd 7.0/7.0	124.5	5.13 dd 6.7/6.7	49.9	
**15α**	131.6		131.6		31.1	1.68 ddd 7.5/11.5/11.5
**15β**						1.46 dd 9.0/11.5
**16α**	17.6	1.63 s	17.8	1.62 s	25.8	2.07 ddd 9.0/14.0/17.0
**16β**						1.60 dd 9.0/14.0
**17**	25.7	1.69 s	25.8	1.68 s	53.0	1.78 dd 9.0/17.0
**18**	24.1	1.16 s	24.2	1.16 s	15.4	0.57 s
**19**	18.7	0.86 s	20.3	1.14 s	15.9	1.06 s
**20**	22.6	1.11 s	25.0	1.33 s	70.4	3.62 dd 6.2/9.0
**21**					22.4	1.21 d 6.2
**22**					15.9	0.90 s
**23**					27.6	1.02 s
**24**					24.9	0.91 s

^a^ Data were measured at 500 MHz for ^1^H and 125 MHz for ^13^C in CDCl_3,_ *δ* in ppm, *J* in Hz.; ^b^ Data were measured at 300 MHz for ^1^H and 75 MHz for ^13^C in CDCl_3_:CD_3_OD = 1:1, *δ* in ppm, *J* in Hz.; ^c^ Overlapping signals were assigned by HSQC, HMBC, COSY, and SELTOCSY experiments.

**Table 2:** Antiproliferative activities of compounds **1**–**3** against two cancer cells and one normal cell line. ^a.^

Cell Line	1	2	3	Etoposide
HepG2	36.3 ± 2.1	64.9 ± 3.5	46.0 ± 2.4	25.4 ± 1.7
HeLa	13.8 ± 1.1	27.1 ± 1.4	12.5 ± 0.9	21.2 ± 1.3
HASMC	>100	>100	>100	63.7

^a^ Results are expressed as IC_50_ values in μM.

## Supplementary Materials

^1^H and ^13^C NMR, ^13^C-DEPT, ^1^H-^13^C HSQC, ^1^H-^13^C HMBC, ^1^H-^1^H COSY, ^1^H-^1^H ROESY, SELTOCSY, UV, and HR-ESI-MS data of compounds **1**–**3** are available in the Supporting Information.
