# Supplementary material for: Prenyleudesmanes and A Hexanorlanostane from the Roots of Lonicera macranthoides
Source: Molecules. 2019 Nov 23;24(23):4276. doi: 10.3390/molecules24234276 (PMC6930473; doi:10.3390/molecules24234276)
Supplement: Supplementary file 1 [file molecules-24-04276-s001.pdf]

Supplementary data

Prenyleudesmanes and a hexanorlanostane from the roots of *Lonicera macranthoides*

Authors

Hui Lyu[1, 2, †], Wenjuan Liu[3, †], Bai Bai[1], Yu Shan[1], Christian Paetz[2], Xu Feng[1, *], Yu Chen[1, *]

Affiliations

[1] Jiangsu Key Laboratory for the Research and Utilization of Plant Resources, The Jiangsu Provincial Platform for Conservation and Utilization of Agricultural Germplasm, Institute of Botany, Jiangsu Province and Chinese Academy of Sciences, Nanjing, China

[2] Max Planck Institute for Chemical Ecology, Jena, Germany

[3] Naval Compound Community Health Care Station, Beijing, China

[*] Correspondence: fengxucnbg@mail.cnbg.ne; Tel.: +86-25-84347158,
yuchen1007@hotmail.com; Tel.: +86-25-84347116

† These two authors contributed equally to this work.

Contents

No.	Content	Page
1	**Figure S1.** HR-ESI-MS spectrum of compound **1**.	S4
2	**Figure S2.** UV spectrum of compound **1**.	S4
3	**Figure S3.** ^{1}H-NMR spectrum (500 MHz, $CDCl_3$) of compound **1**.	S5
4	**Figure S4.** ^{13}C-NMR spectrum (125 MHz, $CDCl_3$) of compound **1**.	S5
5	**Figure S5.** DEPT spectrum (125 MHz, $CDCl_3$) of compound **1**.	S6
6	**Figure S6.** HSQC spectrum (500 MHz, $CDCl_3$) of compound **1**.	S6
7	**Figure S7.** HMBC spectrum (500 MHz, $CDCl_3$) of compound **1**.	S7
8	**Figure S8.** ^{1}H -^{1}H COSY spectrum (500 MHz, $CDCl_3$) of compound **1**.	S7
9	**Figure S9.** ROESY spectrum (500 MHz, $CDCl_3$) of compound **1**.	S8
10	**Figure S10.** SELTOCSY spectrum (500 MHz, transmitter frequency at 1.79 ppm, $CDCl_3$) of compound **1**.	S8
11	**Figure S11.** SELTOCSY spectrum (500 MHz, transmitter frequency at 1.86 ppm, $CDCl_3$) of compound **1**.	S9
12	**Figure S12.** SELTOCSY spectrum (500 MHz, transmitter frequency at 5.14 ppm, $CDCl_3$) of compound **1**.	S9
13	**Figure S13.** HR-ESI-MS spectrum of compound **2**.	S10
14	**Figure S14.** UV spectrum of compound **2**.	S10
15	**Figure S15.** ^{1}H-NMR spectrum (500 MHz, $CDCl_3$) of compound **2**.	S11
16	**Figure S16.** ^{13}C-NMR spectrum (125 MHz, $CDCl_3$) of compound **2**.	S11
17	**Figure S17.** DEPT spectrum (125 MHz, $CDCl_3$) of compound **2**.	S12
18	**Figure S18.** HSQC spectrum (500 MHz, $CDCl_3$) of compound **2**.	S12
19	**Figure S19.** HMBC spectrum (500 MHz, $CDCl_3$) of compound **2**.	S13
20	**Figure S20.** ^{1}H-^{1}H COSY spectrum (500 MHz, $CDCl_3$) of compound **2**.	S13
21	**Figure S21.** ROESY spectrum (500 MHz, $CDCl_3$) of compound **2**.	S14
22	**Figure S22.** SELTOCSY spectrum (500 MHz, transmitter frequency at 4.28 ppm, $CDCl_3$) of compound **2**.	S14
23	**Figure S23.** SELTOCSY spectrum (500 MHz, transmitter	S15

	frequency at 1.88 ppm, $CDCl_3$) of compound **2**.	
24	**Figure S24.** SELTOCSY spectrum (500 MHz, transmitter frequency at 5.13 ppm, $CDCl_3$) of compound **2**.	S15
25	**Figure S25.** HR-ESI-MS spectrum of compound **3**.	S16
26	**Figure S26.** UV spectrum of compound **3**.	S16
27	**Figure S27.** ^{1}H-NMR spectrum (500 MHz, $CDCl_3$:CD_3OD =1:1) of compound **3**.	S17
28	**Figure S28.** ^{13}C-NMR spectrum (125 MHz, $CDCl_3$:CD_3OD =1:1) of compound **3**.	S17
29	**Figure S29.** DEPT spectrum (125 MHz, $CDCl_3$:CD_3OD=1:1) of compound **3**.	S18
30	**Figure S30.** HSQC spectrum (500 MHz, $CDCl_3$:CD_3OD=1:1) of compound **3**.	S18
31	**Figure S31.** HMBC spectrum (500 MHz, $CDCl_3$:CD_3OD=1:1) of compound **3**.	S19
32	**Figure S32.**^{1}H-^{1}H COSY spectrum (500 MHz, $CDCl_3$:CD_3OD =1:1) of compound **3**.	S19
33	**Figure S33.** ROESY spectrum (500 MHz, $CDCl_3$:CD_3OD=1:1) of compound **3**.	S20
34	**Figure S34.** SELTOCSY spectrum (500 MHz, transmitter frequency at 3.57 ppm, $CDCl_3$) of compound **3**.	S20
35	**Figure S35.** SELTOCSY spectrum (500 MHz, transmitter frequency at 5.49 ppm, $CDCl_3$) of compound **3**.	S21
36	**Figure S36.** SELTOCSY spectrum (500 MHz, transmitter frequency at 6.31 ppm, $CDCl_3$) of compound **3**.	S21
37	**Figure S37.** SELTOCSY spectrum (500 MHz, transmitter frequency at 3.62 ppm, $CDCl_3$) of compound **3**.	S22

Qualitative Analysis Report

**Data Filename**	LH-4216-LMB-15.d	**Sample Name**	LH-4216
**Sample Type**	Sample	**Position**	P2-D3
**Instrument Name**	Instrument 1	**User Name**	
**Acq Method**	LH LML.m	**Acquired Time**	4/14/2018 10:35:59 AM
**IRM Calibration Status**	Success	**DA Method**	YINMIN.m
**Comment**			

Sample Group **Info.**

User Spectra

Fragmentor Voltage	Collision Energy	Ionization Mode
50	0	ESI

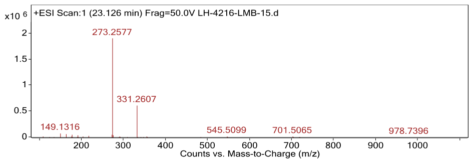

Peak List

m/z	z	Abund
149.1316	1	81138.58
163.1475	1	71275.75
177.1631	1	56453.14
271.2417	1	61072.92
273.2577	1	1908992.13
273.3995	1	119781.39
273.4571	2	71117.74
274.2607	1	484225.53
331.2607	1	616329.88
332.2648	1	126626.41

Agilent Technologies   Page 1 of 1   Printed at: 10:37 AM on: 4/18/2018

Figure S1. HR-ESI-MS spectrum of compound **1**.

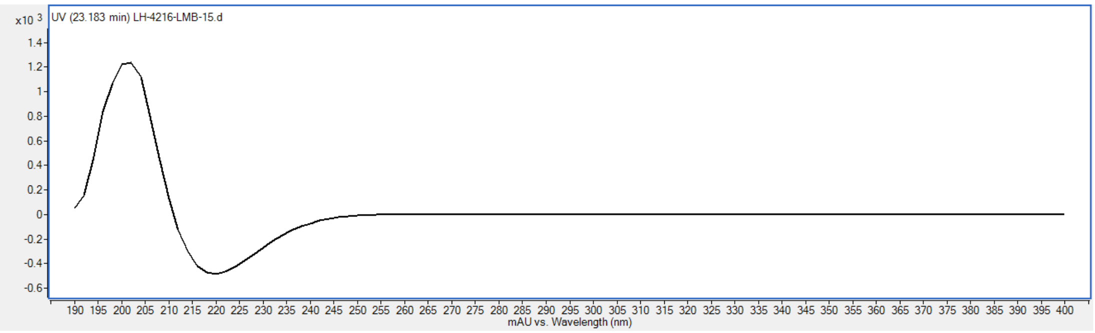

Figure S2. UV spectrum of compound **1**.

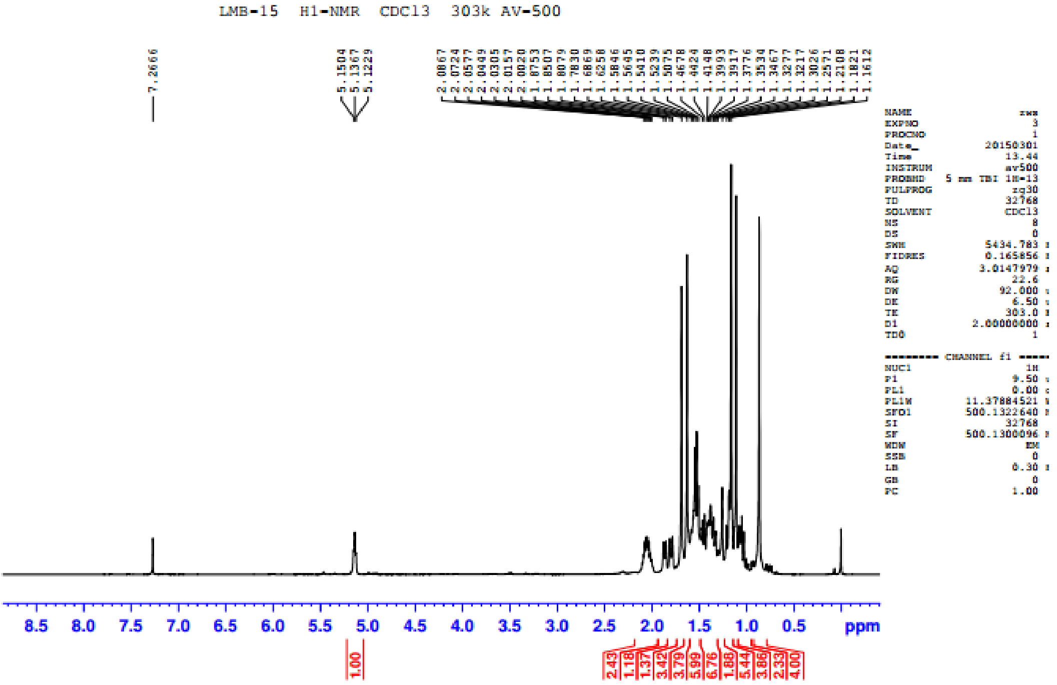

Figure S3. ^{1}H-NMR spectrum (500 MHz, $CDCl_3$) of compound **1**.

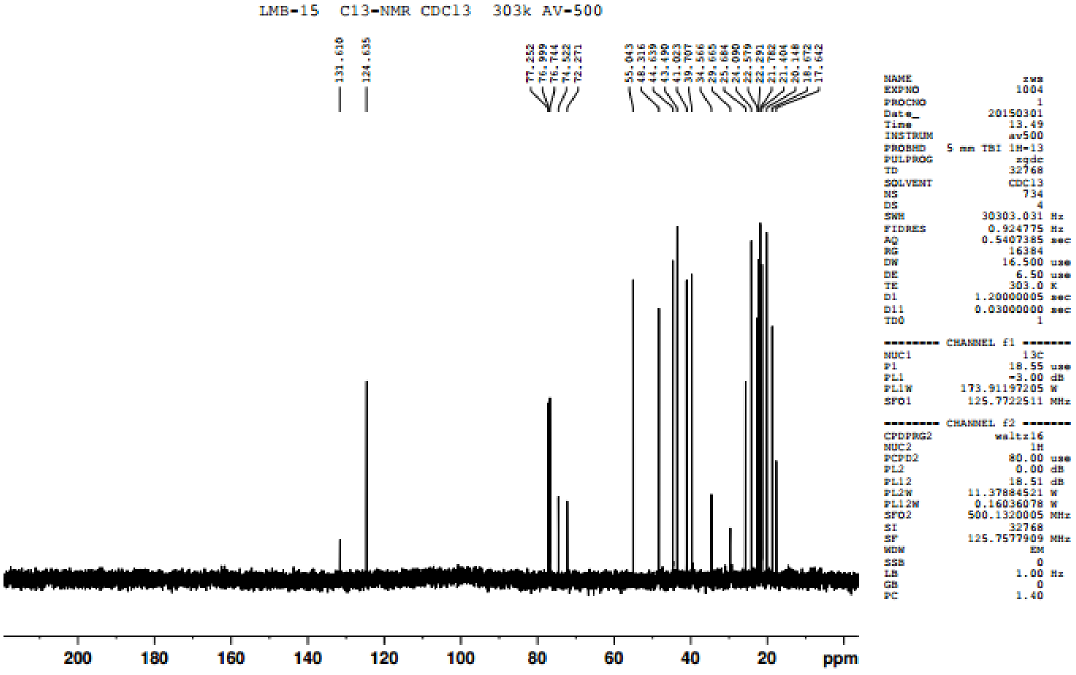

Figure S4. ^{13}C-NMR spectrum (125 MHz, $CDCl_3$) of compound **1**.

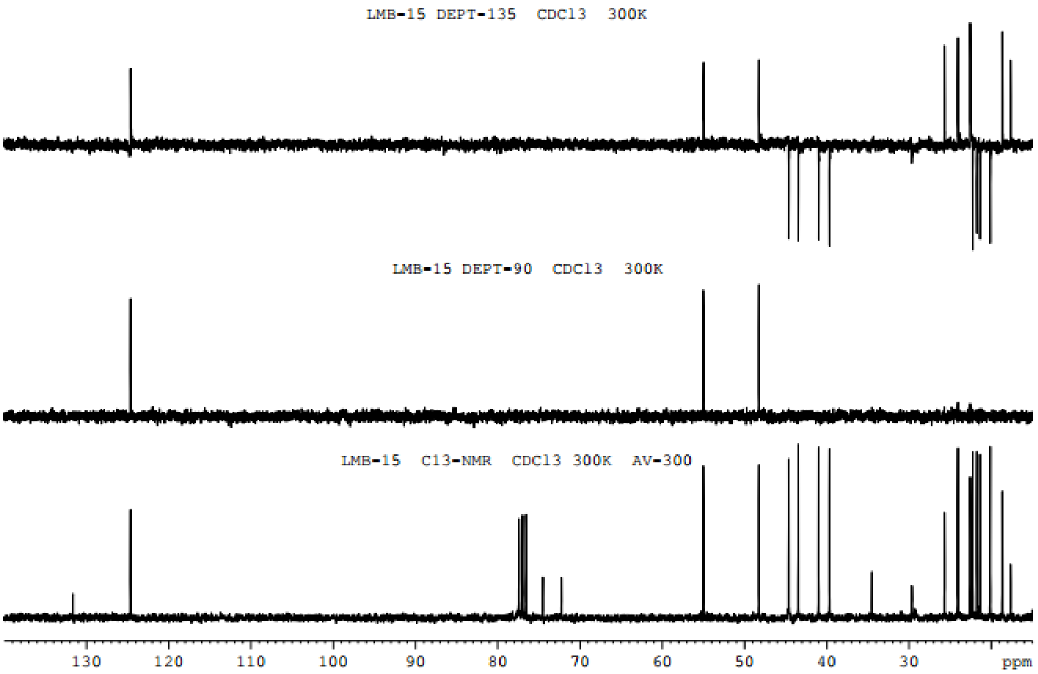

Figure S5. DEPT spectrum (125 MHz, $CDCl_3$) of compound **1**.

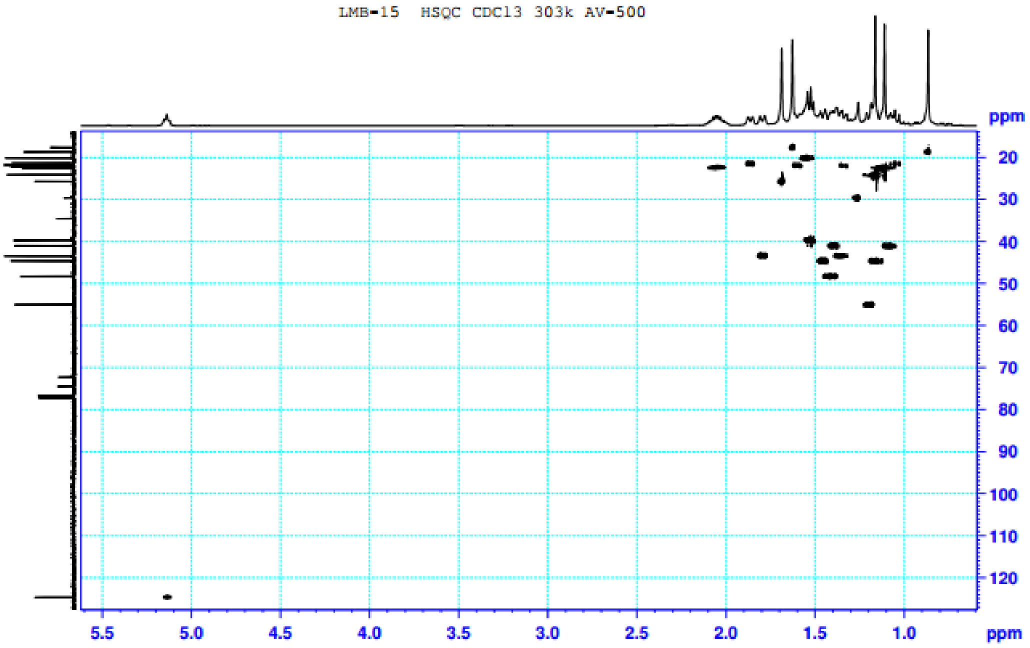

Figure S6. HSQC spectrum (500 MHz, $CDCl_3$) of compound **1**.

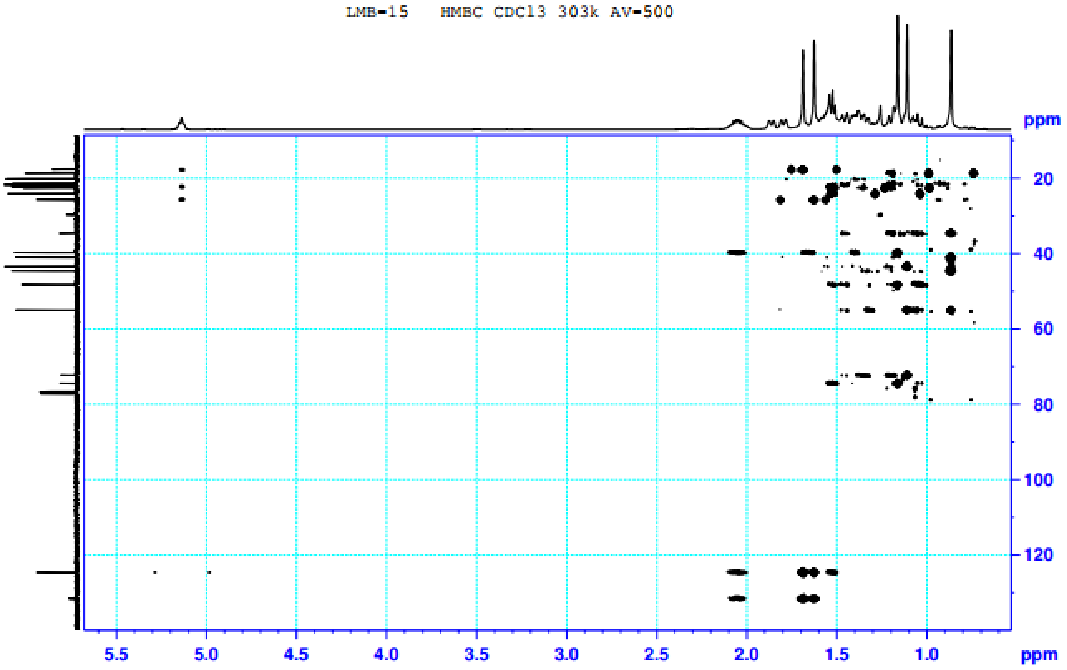

Figure S7. HMBC spectrum (500 MHz, $CDCl_3$) of compound **1**.

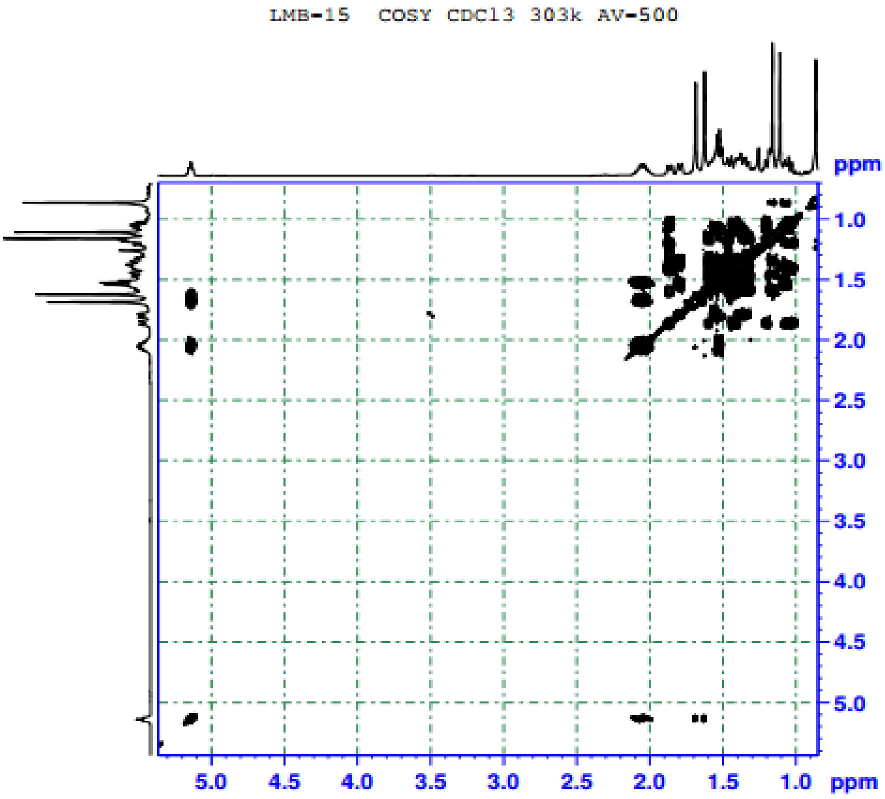

Figure S8. ^{1}H-^{1}H COSY spectrum (500 MHz, $CDCl_3$) of compound **1**.

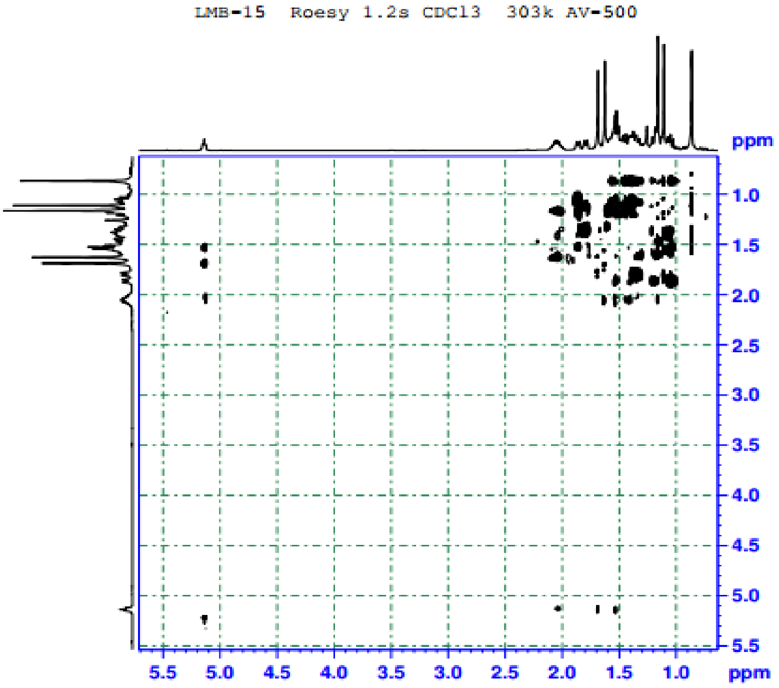

Figure S9. ROESY spectrum (500 MHz, $CDCl_3$) of compound **1**.

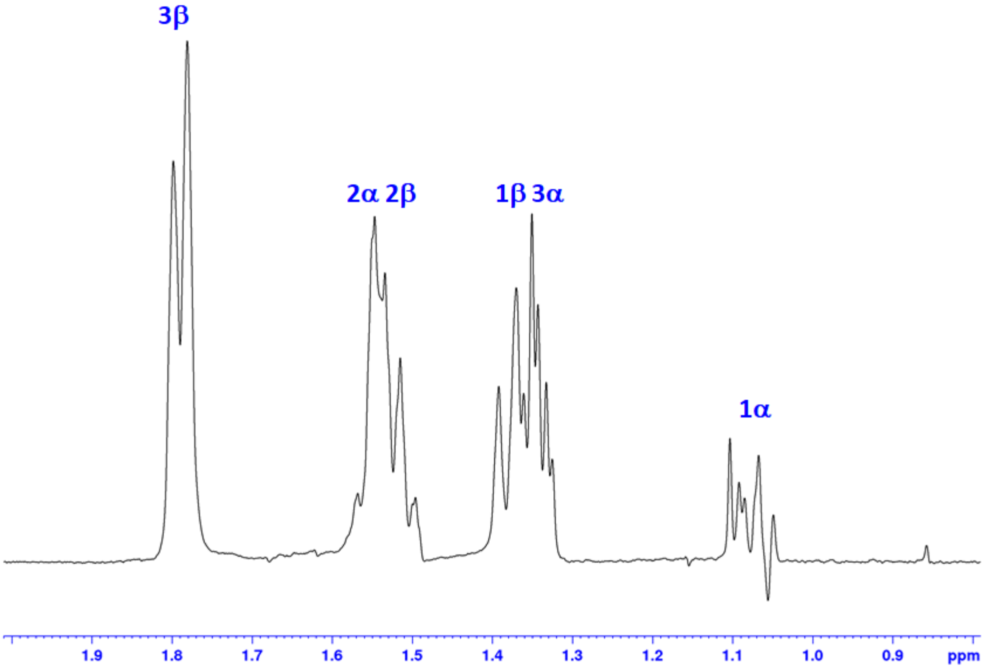

Figure S10. SELTOCSY spectrum (500 MHz, transmitter frequency at 1.79 ppm, 10 Hz width, 120 ms mix time, $CDCl_3$) of compound **1**.

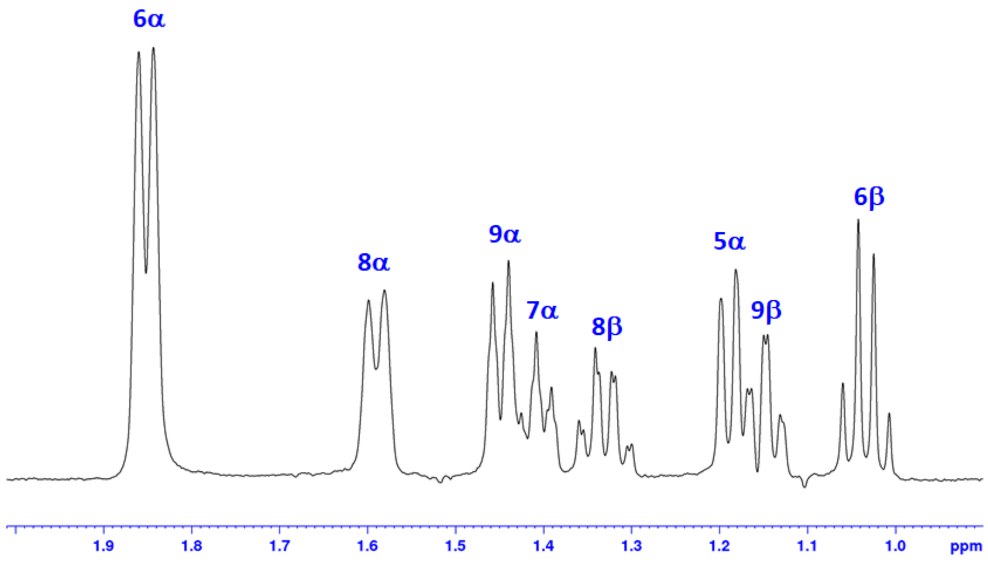

Figure S11. SELTOCSY spectrum (500 MHz, transmitter frequency at 1.86 ppm, 10 Hz width, 120 ms mix time, $CDCl_3$) of compound **1**.

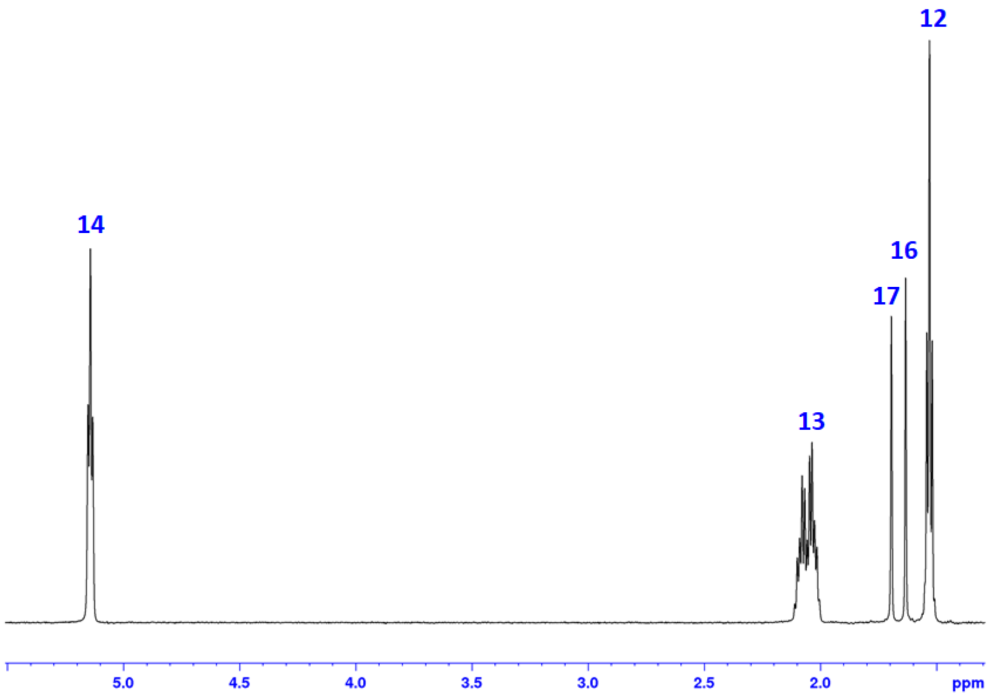

Figure S12. SELTOCSY spectrum (500 MHz, transmitter frequency at 5.14 ppm, 10 Hz width, 120 ms mix time, $CDCl_3$) of compound **1**.

Qualitative Analysis Report

**Data Filename**	LH-4214-LMR-9.d	**Sample Name**	LH-4214
**Sample Type**	Sample	**Position**	P2-D1
**Instrument Name**	Instrument 1	**User Name**	
**Acq Method**	LH LML.m	**Acquired Time**	4/14/2018 8:54:10 AM
**IRM Calibration Status**	Success	**DA Method**	YINMIN.m
**Comment**			

Sample Group **Info.**

User Spectra

Fragmentor Voltage	Collision Energy	Ionization Mode
200	0	ESI

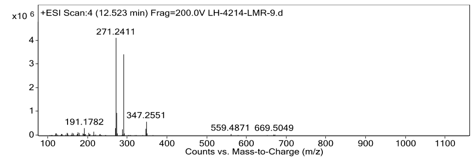

Peak List

m/z	z	Abund
191.1782	1	313494.78
269.2259	1	320340.78
271.2411	2	4123874.75
271.3824	1	277907.56
272.2442	2	973332.25
287.2361	1	269770.91
289.2518	1	3439059.5
290.2548	1	816644.81
345.2397	1	285323.72
347.2551	1	593094.56

Agilent Technologies   Page 1 of 1   Printed at: 10:18 AM on: 4/18/2018

Figure S13. HR-ESI-MS spectrum of compound **2**.

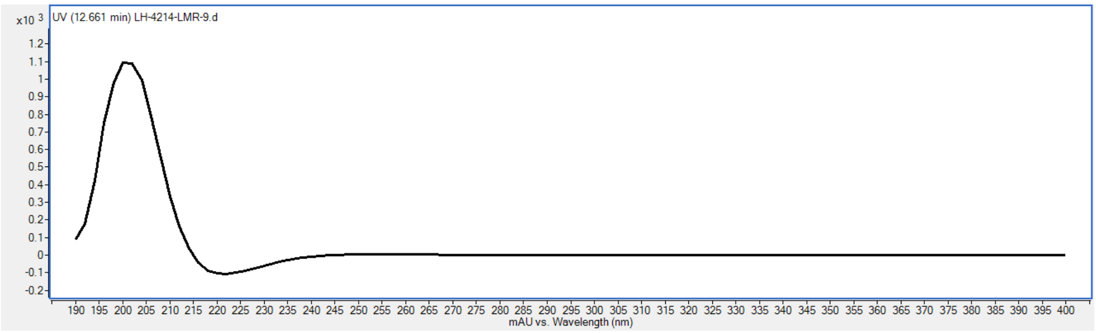

Figure S14. UV spectrum of compound **2**.

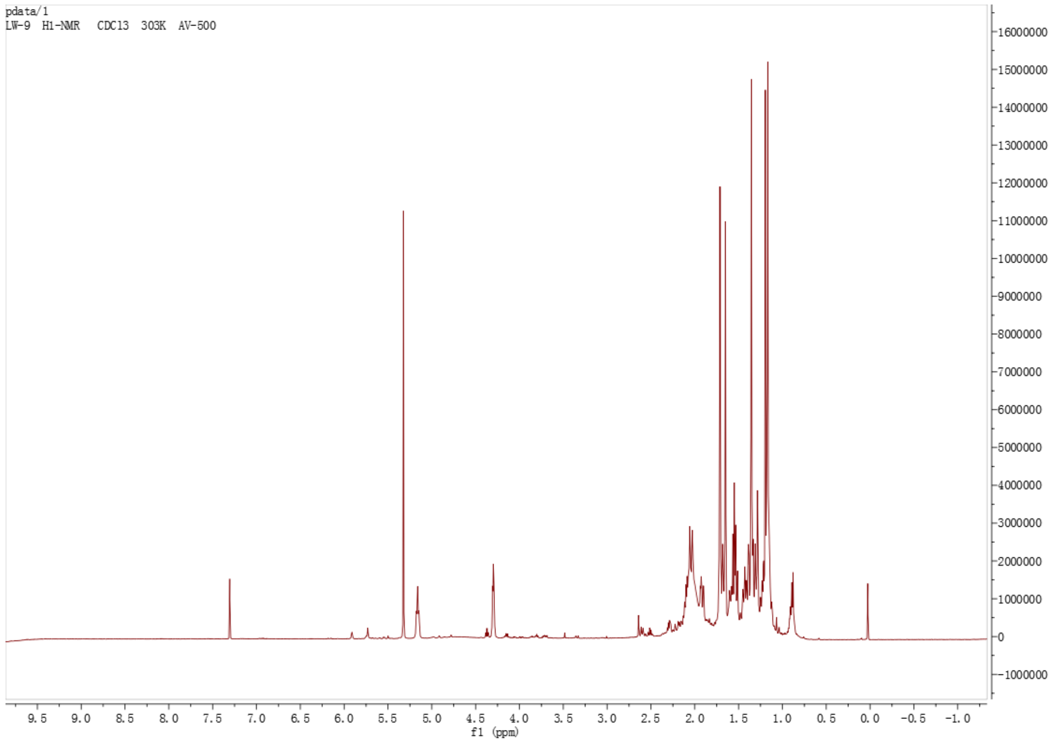

Figure S15. ^{1}H-NMR spectrum (500 MHz, $CDCl_3$) of compound **2**.

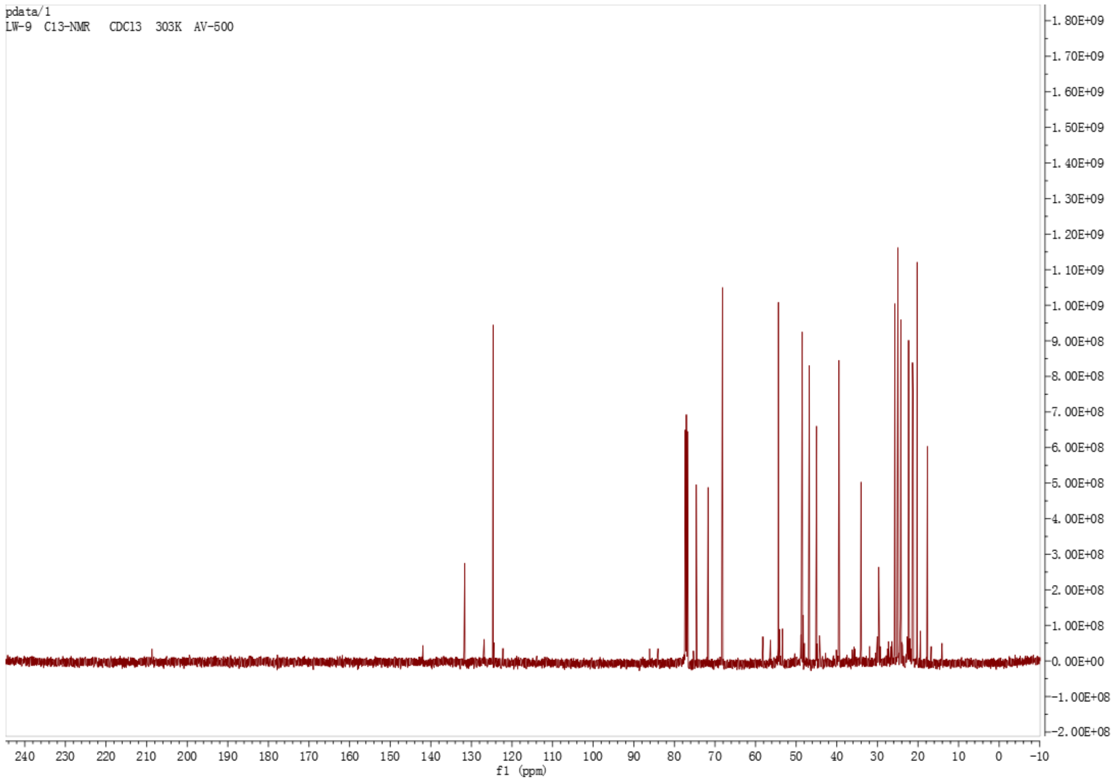

Figure S16. ^{13}C-NMR spectrum (125 MHz, $CDCl_3$) of compound **2**.

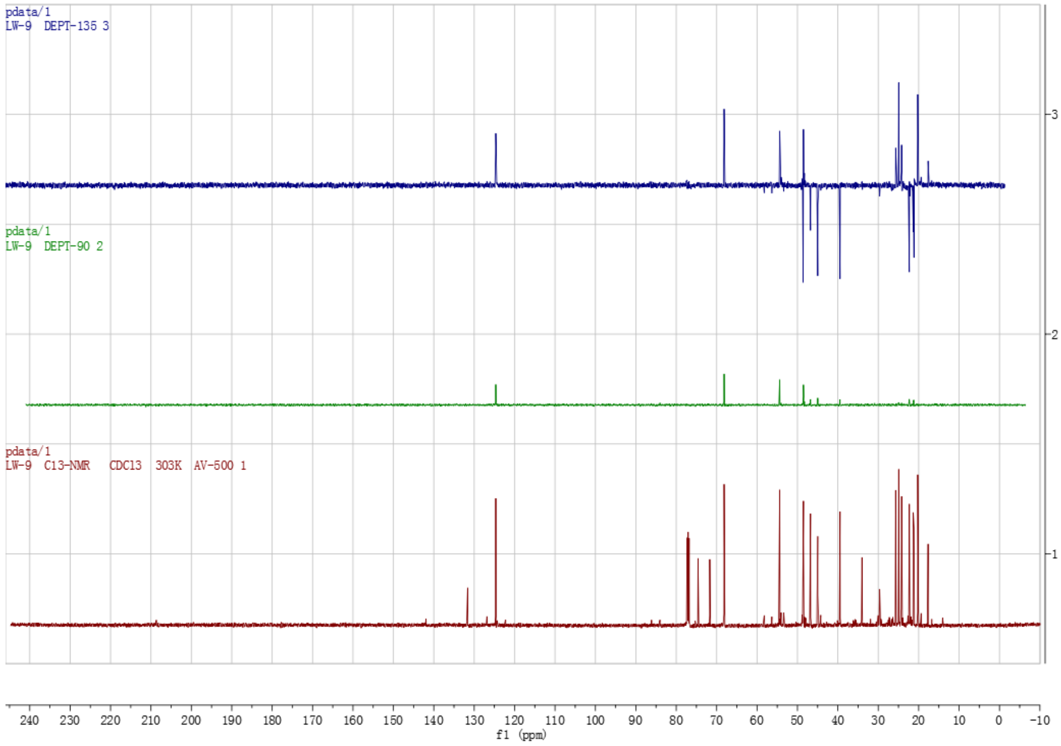

Figure S17. DEPT spectrum (125 MHz, $CDCl_3$) of compound **2**.

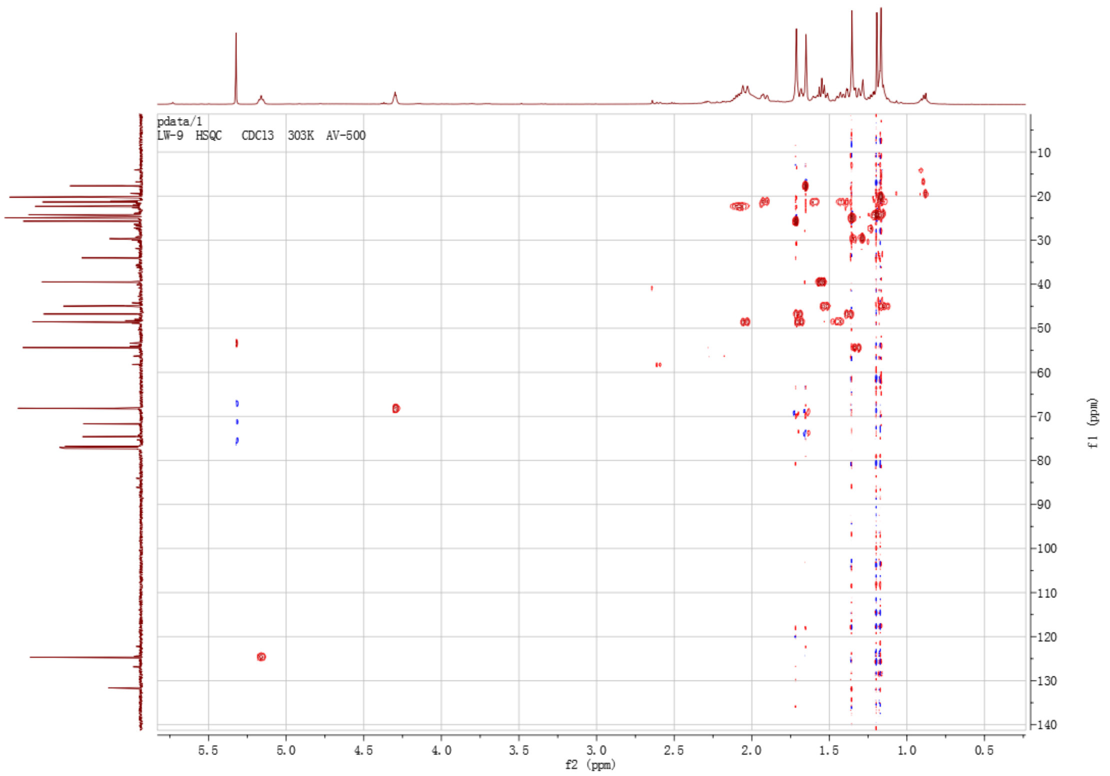

Figure S18. HSQC spectrum (500 MHz, $CDCl_3$) of compound **2**.

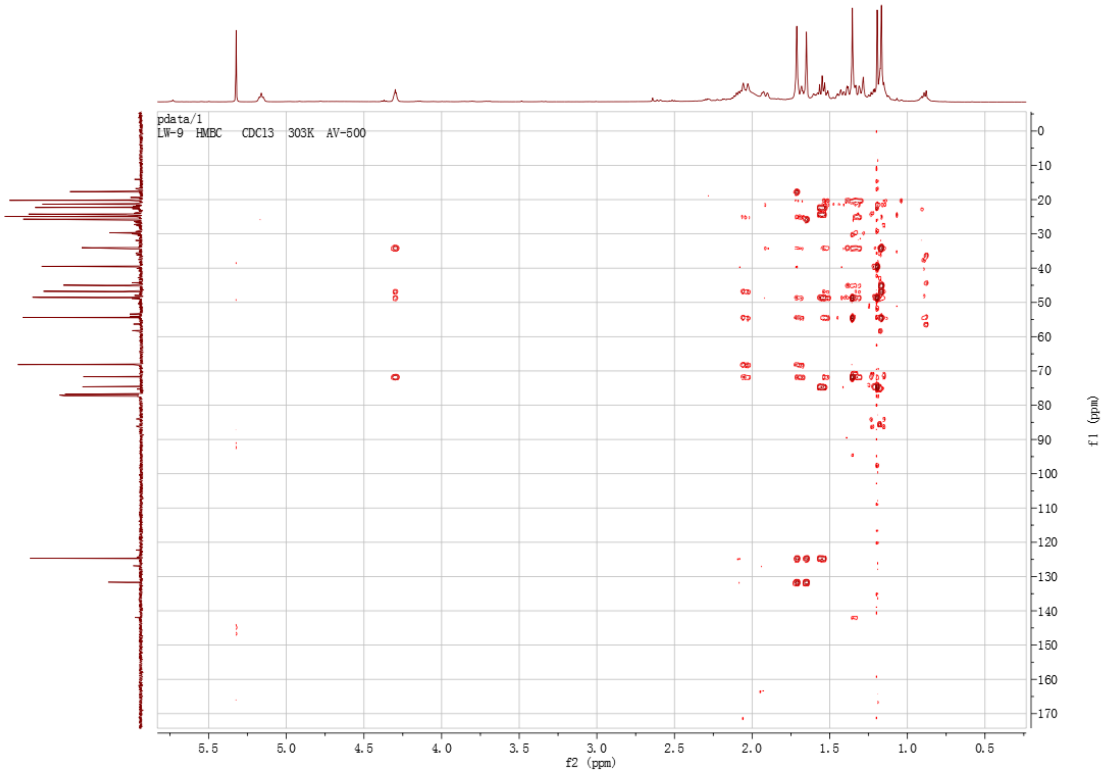

Figure S19. HMBC spectrum (500 MHz, $CDCl_3$) of compound **2**.

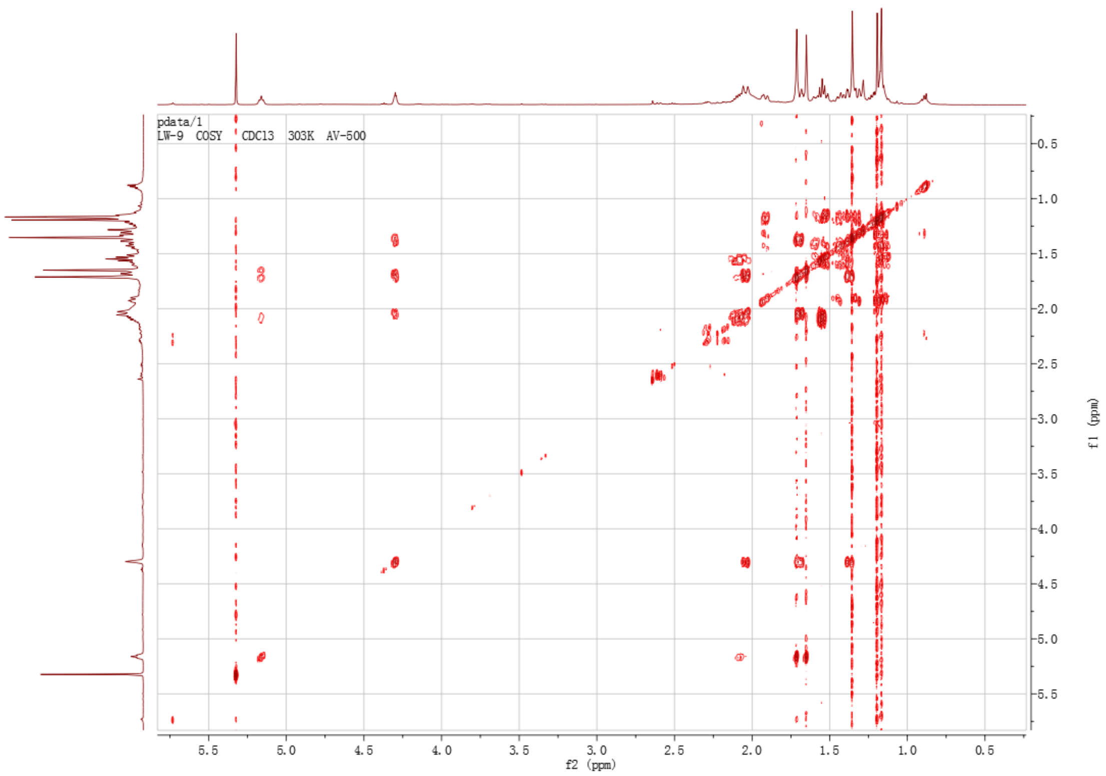

Figure S20. ^1H-^1H COSY spectrum (500 MHz, $CDCl_3$) of compound **2**.

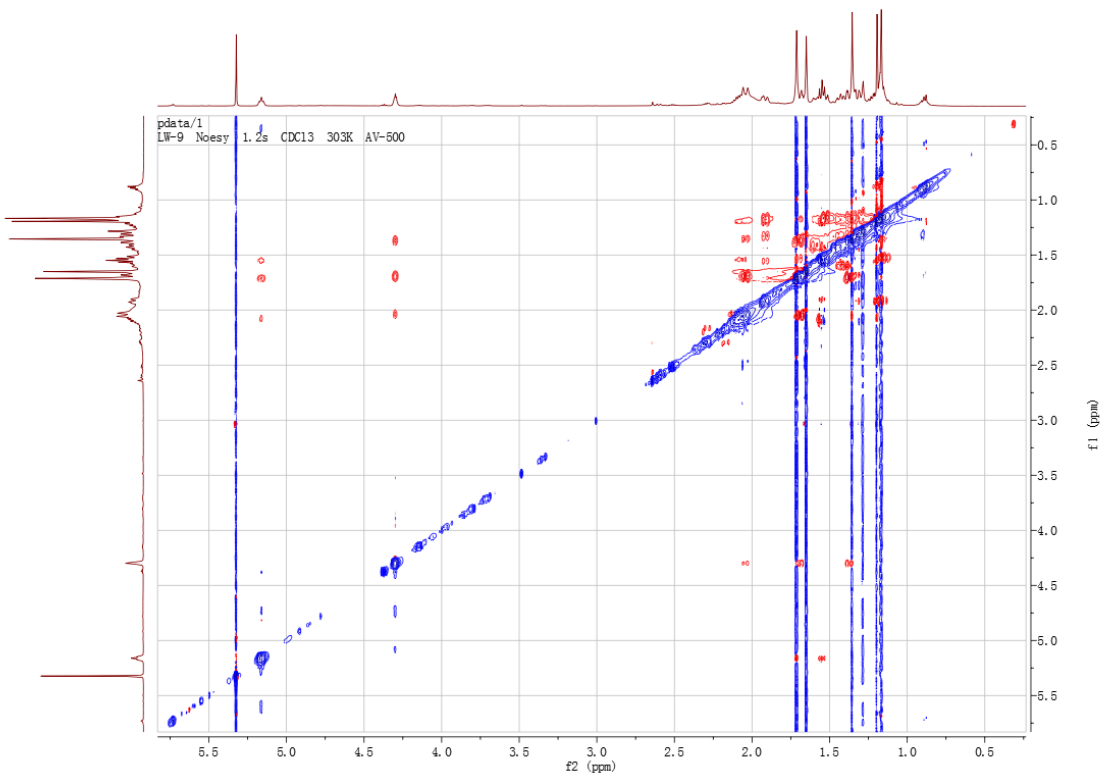

Figure S21. ROESY spectrum (500 MHz, $CDCl_3$) of compound **2**.

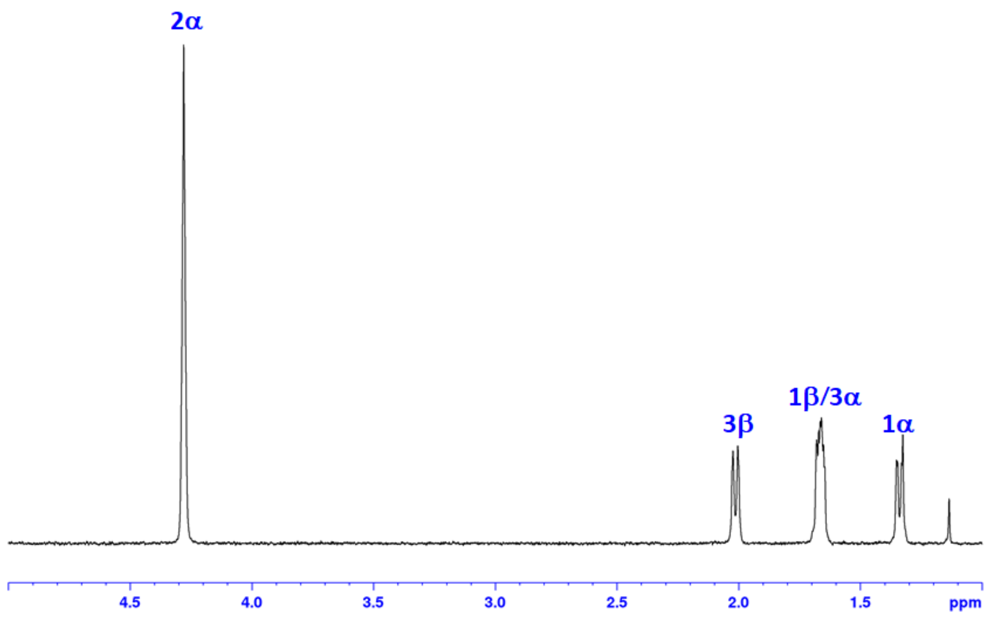

Figure S22. SELTOCSY spectrum (500 MHz, transmitter frequency at 4.28 ppm, 10 Hz width, 120 ms mix time, $CDCl_3$) of compound **2**.

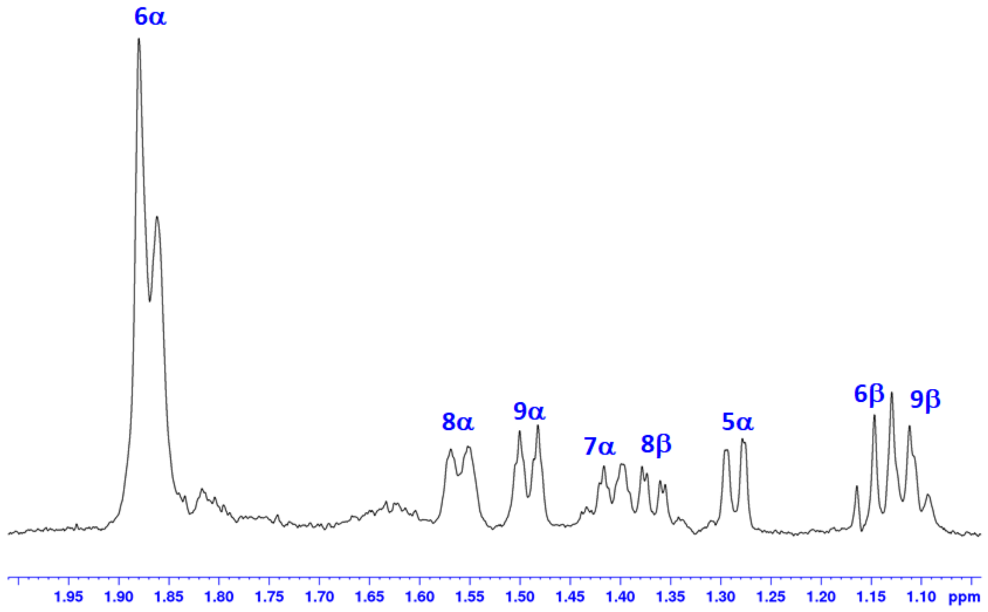

Figure S23. SELTOCSY spectrum (500 MHz, transmitter frequency at 1.88 ppm, 10 Hz width, 120 ms mix time, $CDCl_3$) of compound **2**.

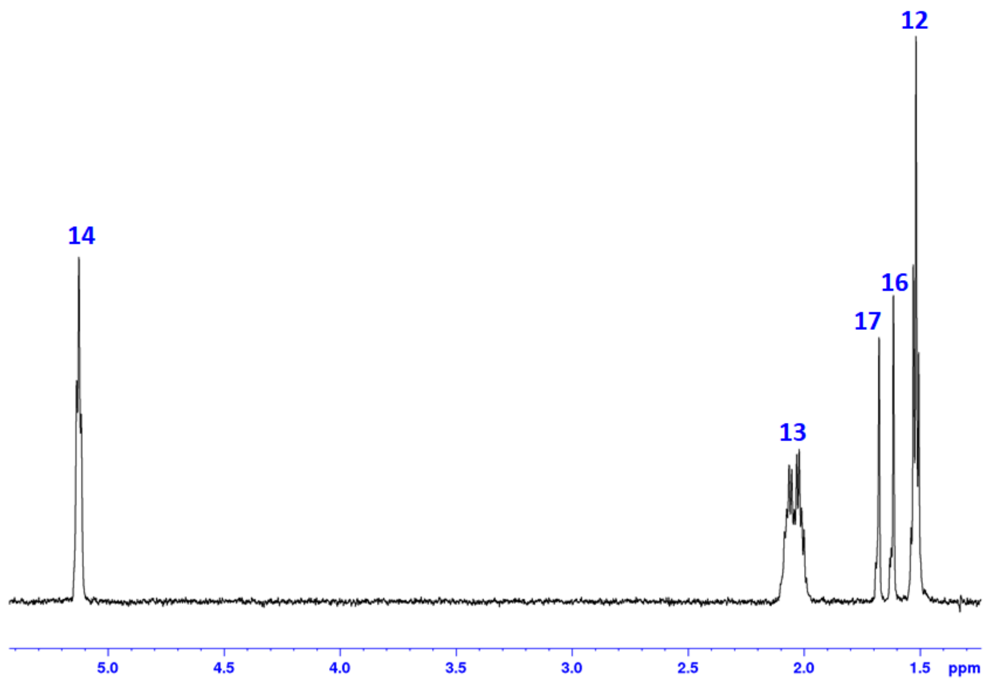

Figure S24. SELTOCSY spectrum (500 MHz, transmitter frequency at 5.13 ppm, 10 Hz width, 120 ms mix time, $CDCl_3$) of compound **2**.

Qualitative Analysis Report

**Data Filename**	LH-4215-LMR-10.d	**Sample Name**	LH-4215
**Sample Type**	Sample	**Position**	P2-D2
**Instrument Name**	Instrument 1	**User Name**	
**Acq Method**	LH LML.m	**Acquired Time**	4/14/2018 9:45:06 AM
**IRM Calibration Status**	Success	**DA Method**	YINMIN.m
**Comment**			

Sample Group **Info.**

User Spectra

Fragmentor Voltage	Collision Energy	Ionization Mode
200	0	ESI

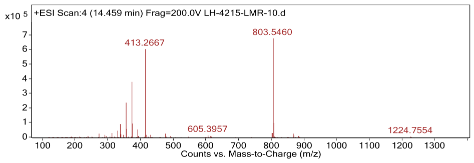

Peak List

m/z	z	Abund
337.2523	1	91990.57
355.2631	1	239436.2
356.2673	1	60103.9
373.2743	1	380411.75
374.2769	1	95701.25
413.2667	1	606927.63
414.2702	1	140741.06
803.546	1	679692.56
804.5512	1	347174.38
805.5525	1	101199.11

Agilent Technologies Page 1 of 1 Printed at: 10:28 AM on: 4/18/2018

Figure S25. HR-ESI-MS spectrum of compound **3**.

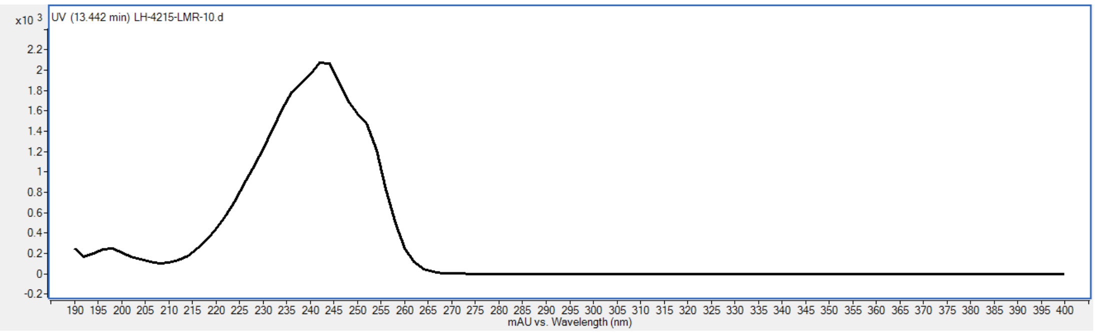

Figure S26. UV spectrum of compound **3**.

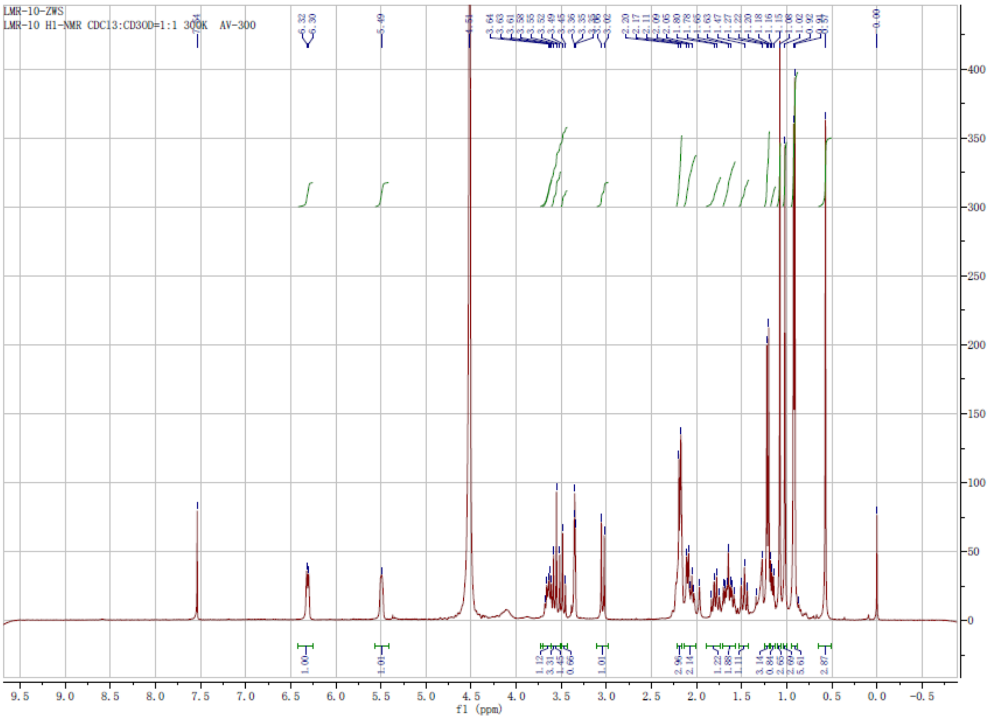

Figure S27. ^{1}H-NMR spectrum (500 MHz, $CDCl_3$:CD_3OD=1:1) of compound **3**.

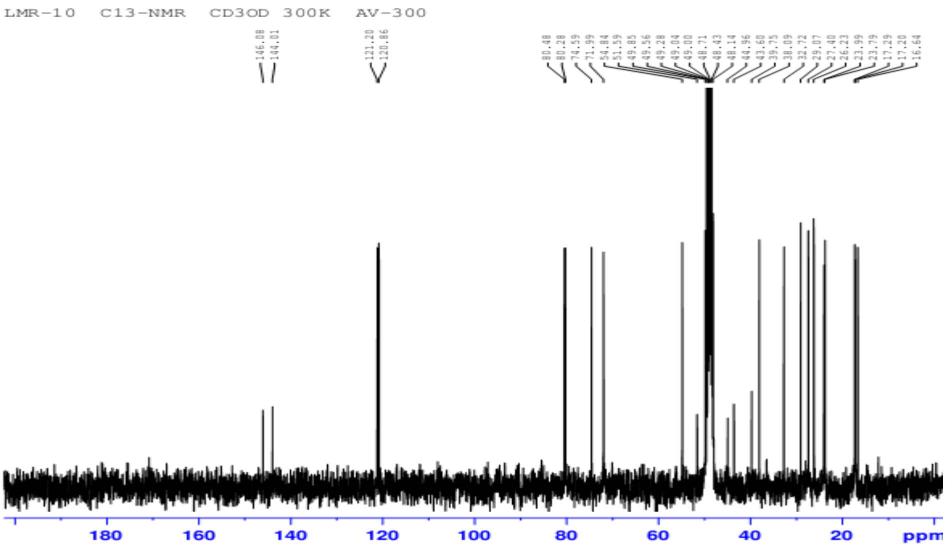

Figure S28. ^{13}C-NMR spectrum (125 MHz, $CDCl_3$:CD_3OD=1:1) of compound **3**.

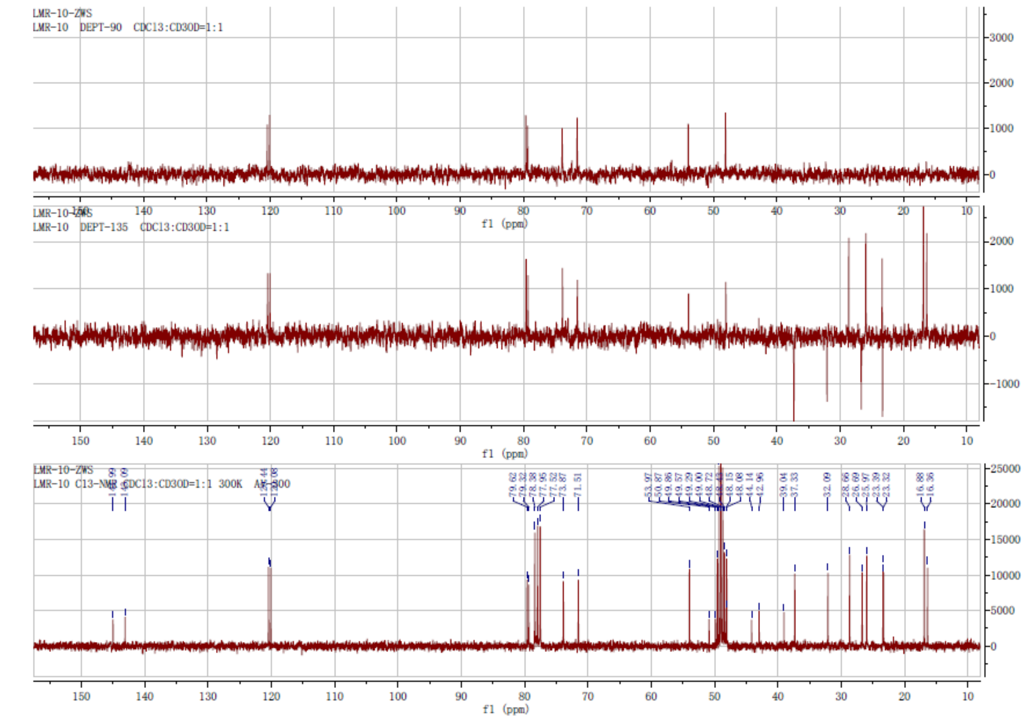

Figure S29. DEPT spectrum (125 MHz, $CDCl_3$:CD_3OD=1:1) of compound **3**.

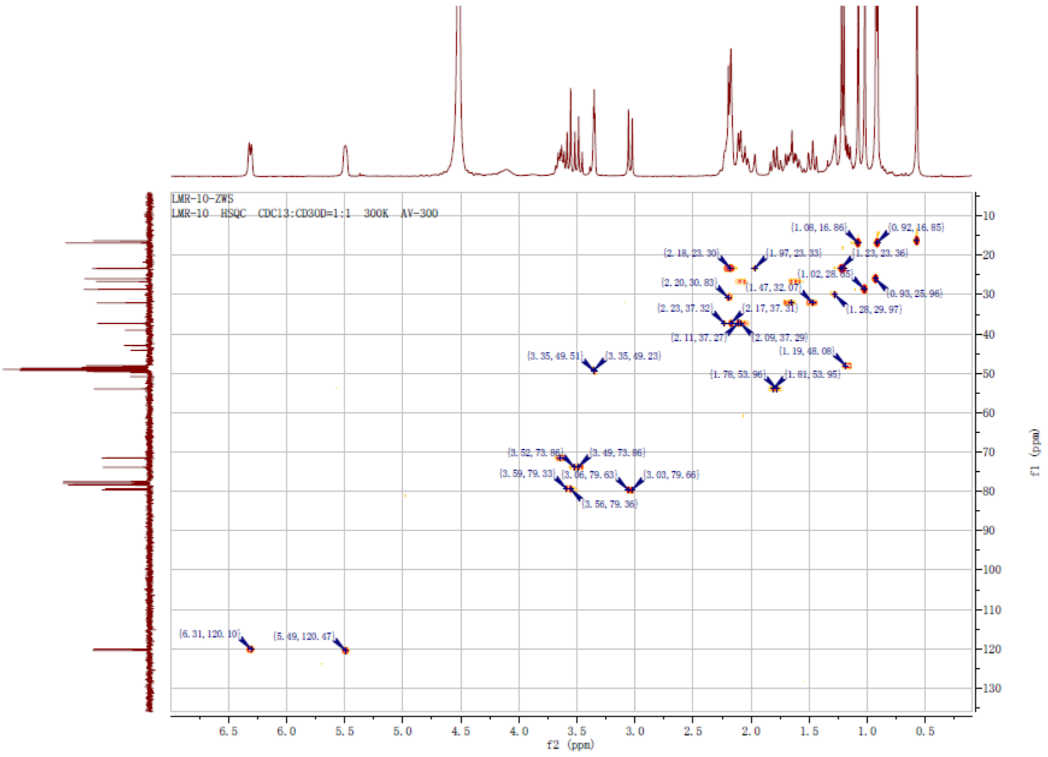

Figure S30. HSQC spectrum (500 MHz, $CDCl_3$:CD_3OD=1:1) of compound **3**.

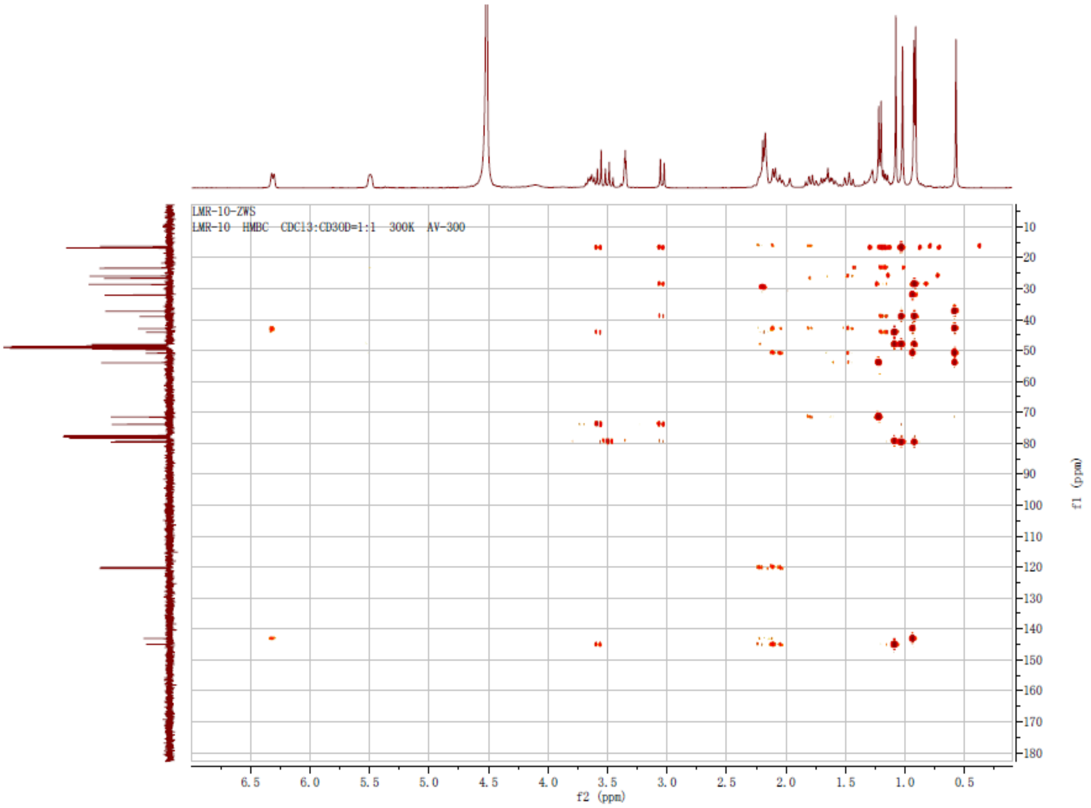

Figure S31. HMBC spectrum (500 MHz, $CDCl_3$:CD_3OD=1:1) of compound **3**.

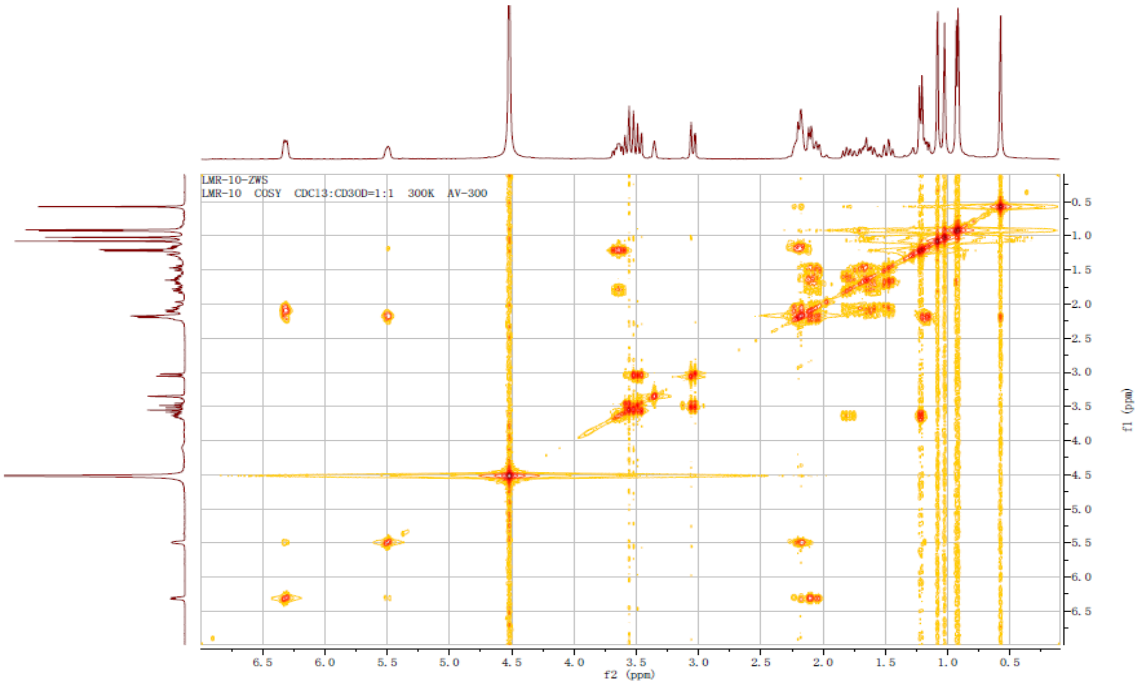

Figure S32. ^1H-^1H COSY spectrum (500 MHz, $CDCl_3$:CD_3OD=1:1) of compound **3**.

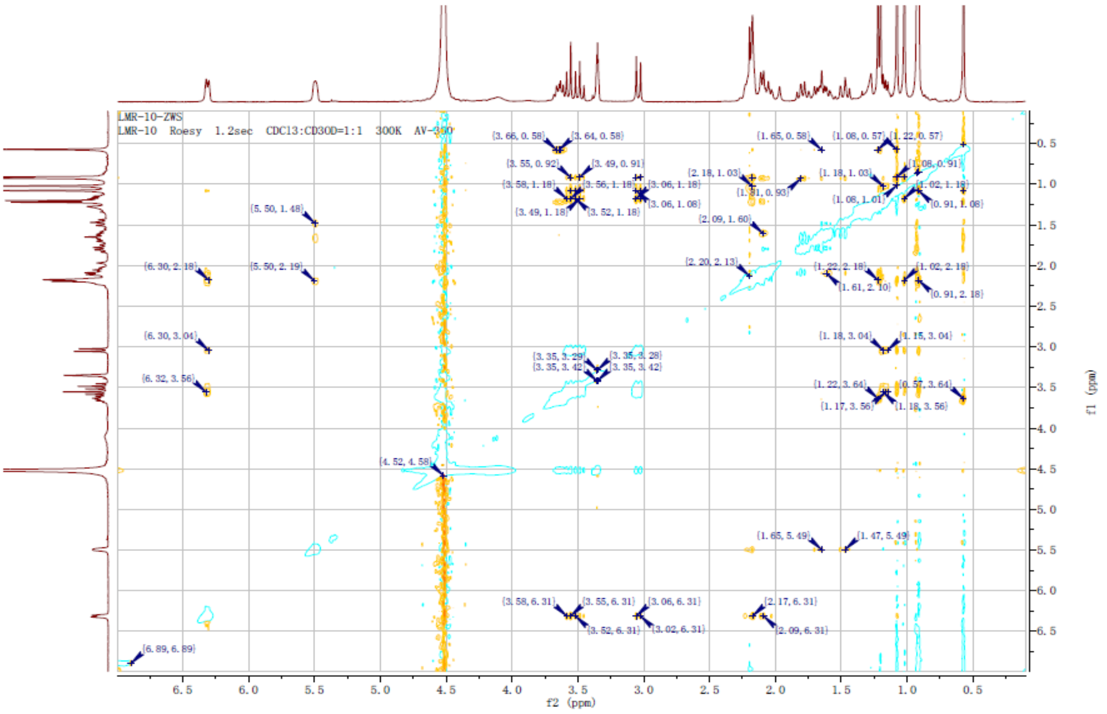

Figure S33. ROESY spectrum (500 MHz, $CDCl_3$:CD_3OD=1:1) of compound **3**.

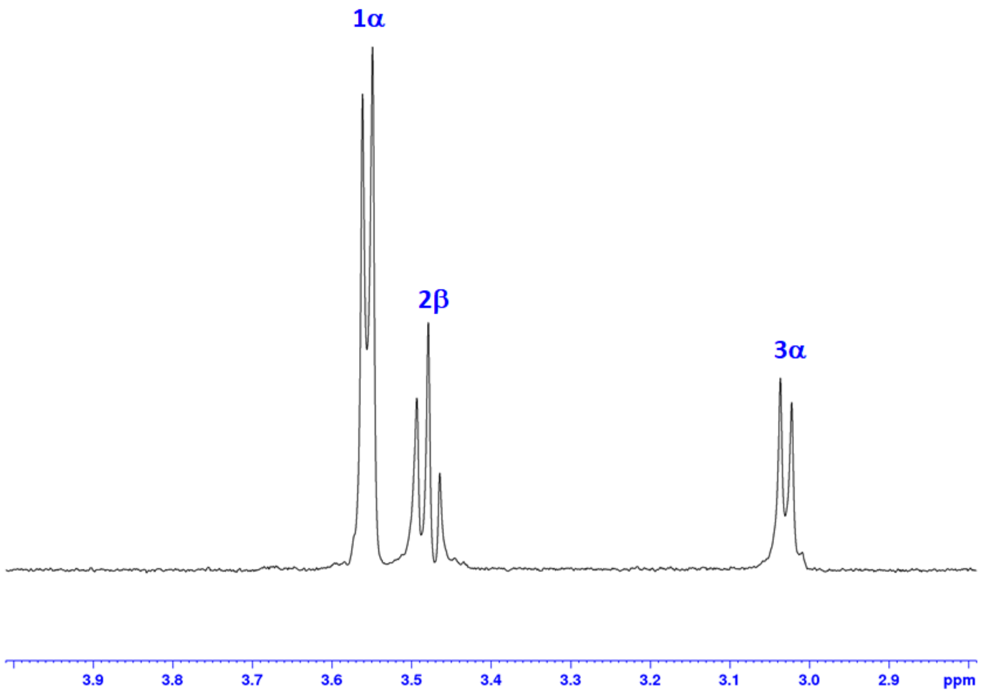

Figure S34. SELTOCSY spectrum (500 MHz, transmitter frequency at 3.57 ppm, 10 Hz width, 120 ms mix time, $CDCl_3$) of compound **3**.

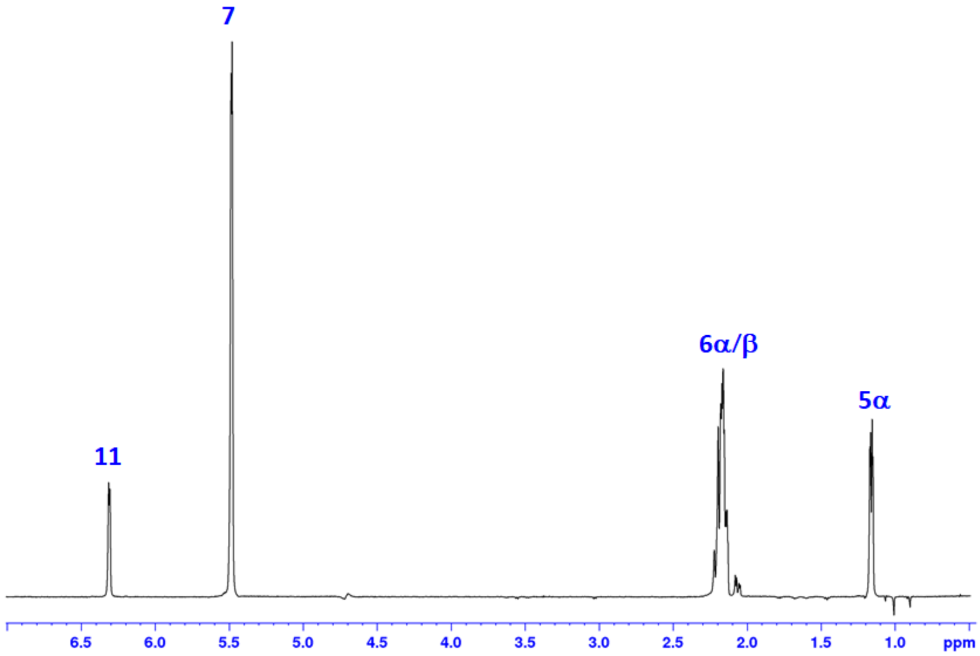

Figure S35. SELTOCSY spectrum (500 MHz, transmitter frequency at 5.49 ppm, 10 Hz width, 120 ms mix time, $CDCl_3$) of compound **3**.

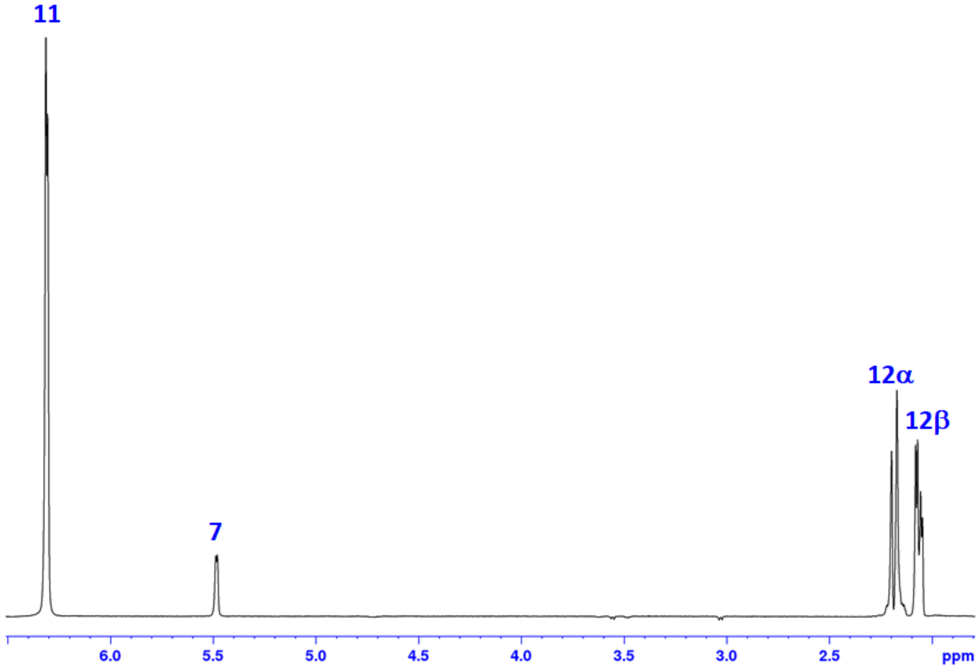

Figure S36. SELTOCSY spectrum (500 MHz, transmitter frequency at 6.31 ppm, 10 Hz width, 120 ms mix time, $CDCl_3$) of compound **3**.

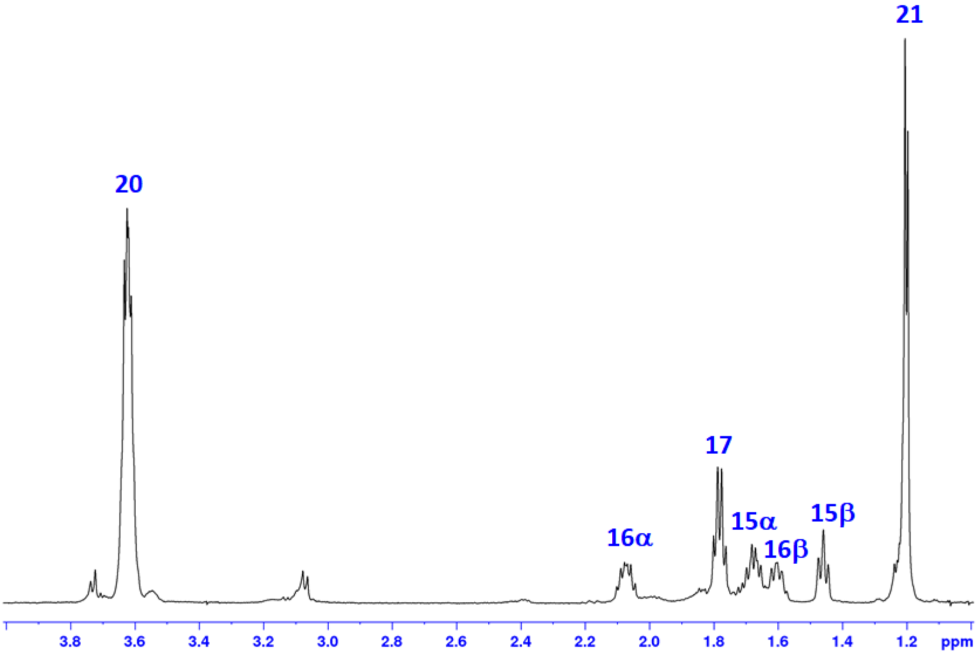

Figure S37. SELTOCSY spectrum (500 MHz, transmitter frequency at 3.62 ppm, 10 Hz width, 120 ms mix time, $CDCl_3$) of compound **3**.
